# Solvent-vapour-assisted pathways and the role of pre-organization in solid-state transformations of coordination polymers

**DOI:** 10.1107/S2052252515000147

**Published:** 2015-02-26

**Authors:** James S. Wright, Iñigo J. Vitórica-Yrezábal, Harry Adams, Stephen P. Thompson, Adrian H. Hill, Lee Brammer

**Affiliations:** a Department of Chemistry, University of Sheffield, Brook Hill, Sheffield S3 7HF, UK; b Diamond Light Source, Harwell Science and Innovation Campus, Didcot, Oxfordshire OX11 0DE, UK; c European Synchrotron Radiation Facility, 6 rue J. Horowitz, 38042 Grenoble, France

**Keywords:** solid-state transformation, coordination polymers, solvent-vapour-assisted conversion

## Abstract

A family of one-dimensional coordination polymers, [Ag_4_(O_2_C(CF_2_)_2_CF_3_)_4_(phenazine)_2_(arene)_*n*_]·*m*(arene), **1** (arene = toluene or xylene), have been synthesized and crystallographically characterized. Arene guest loss invokes structural transformations to yield a pair of polymorphic coordination polymers [Ag_4_(O_2_C(CF_2_)_2_CF_3_)_4_(phenazine)_2_], **2*a*** and/or **2*b***, with one- and two-dimensional architectures, respectively. The role of pre-organization of the polymer chains of **1** in the selectivity for formation of either polymorph is explored, and the templating effect of toluene and *p*-xylene over *o*-xylene or *m*-xylene in the formation of arene-containing architecture **1** is also demonstrated. The formation of arene-free phase **2*b***, not accessible in a phase-pure form through other means, is shown to be the sole product of loss of toluene from **1-tol·tol** [Ag_4_(O_2_C(CF_2_)_2_CF_3_)_4_(phenazine)_2_(toluene)]·2(toluene), a phase containing toluene coordinated to Ag(I) in an unusual μ:η^1^,η^1^ manner. Solvent-vapour-assisted conversion between the polymorphic coordination polymers and solvent-vapour influence on the conversion of coordination polymers **1** to **2*a*** and **2*b*** is also explored. The transformations have been examined and confirmed by X-ray diffraction, NMR spectroscopy and thermal analyses, including *in situ* diffraction studies of some transformations.

## Abstract 2

The structural transformations of a family of coordination polymers that entrap small arenes (toluene, xylene) by Ag(I) coordination has been investigated. The study highlights the role in these transformations of vapour–solid interactions and pre-organization in the solid state.

## 1. Introduction   

Designed solid-state materials are of increasing interest, an important class of which is coordination polymers, in which metal ions or clusters are connected by organic ligands (linkers) to create extended network solids that are periodic and usually crystalline. Porous coordination polymers (PCPs), more commonly known as metal–organic frameworks (MOFs), have enjoyed particular attention due to their potential application in gas sorption and separation (Li *et al.*, 1999 ▶; Zhang & Chen, 2009 ▶; Sumida *et al.*, 2009 ▶; D’Alessandro *et al.*, 2010 ▶; Burd *et al.*, 2012 ▶; FitzGerald *et al.*, 2013 ▶; Huang *et al.*, 2013 ▶; Carrington *et al.*, 2014 ▶), heterogeneous catalysis (Gomez-Lor *et al.*, 2002 ▶; Wu *et al.*, 2005 ▶; Lee *et al.*, 2009 ▶; Li *et al.*, 2009 ▶, 2014 ▶) and novel optical and magnetic properties (Evans & Lin, 2002 ▶; Zhou *et al.*, 2013 ▶; Baldoví *et al.*, 2014 ▶; Wang *et al.*, 2014 ▶). The post-synthetic modification (PSM) of coordination polymers and PCPs has only more recently been the focus of more detailed work (Ingleson *et al.*, 2008 ▶; Tanabe *et al.*, 2008 ▶; Wang & Cohen, 2009 ▶; Nguyen & Cohen, 2010 ▶; Vermeulen *et al.*, 2013 ▶; Zheng *et al.*, 2013 ▶; Li *et al.*, 2013 ▶), facilitating the multi-step synthesis of materials (Ingleson *et al.*, 2008 ▶; Tanabe *et al.*, 2008 ▶; Wang & Cohen, 2009 ▶; Nguyen & Cohen, 2010 ▶; Vermeulen *et al.*, 2013 ▶; Zheng *et al.*, 2013 ▶; Li *et al.*, 2013 ▶; Libri *et al.*, 2008 ▶; Vitórica-Yrezábal *et al.*, 2013 ▶), stereo- or regio-selective transformation of ligands (Jones & Bauer, 2009 ▶) or the modification of solid-state properties of porous materials (Wang & Cohen, 2009 ▶; Nguyen & Cohen, 2010 ▶). The flexibility and responsiveness of some MOFs to removal of non-covalently bound solvent or uptake of gas molecules has been described by many groups and was highlighted by Kitagawa and co-workers at an early stage in their 2004 review in which they classified MOFs into first-, second- and third-generation materials, the latter being materials which could undergo structural changes and recover pore integrity as well as retaining or recovering crystallinity during such guest loss/uptake processes (Kitagawa *et al.*, 2004 ▶).

More generally, the reactions and structural transformations of coordination polymers include a variety of transformations involving solvation or desolvation processes, typically involving water, alcohols or acetonitrile as the solvent molecule. Such transformations involving coordinated solvent molecules can result in changes in coordination environment at the metal centres wherein terminally coordinated solvent molecules are replaced by bridging (linker) ligands during desolvation (and *vice versa* during solvent uptake). Reviews by Kole & Vittal (2013 ▶) and by Li & Du (2011 ▶) consider transformations of this type along with other solid-state transformations, including photochemical transformations and transformations induced by input of heat or mechanochemical energy.

**Figure 1:**
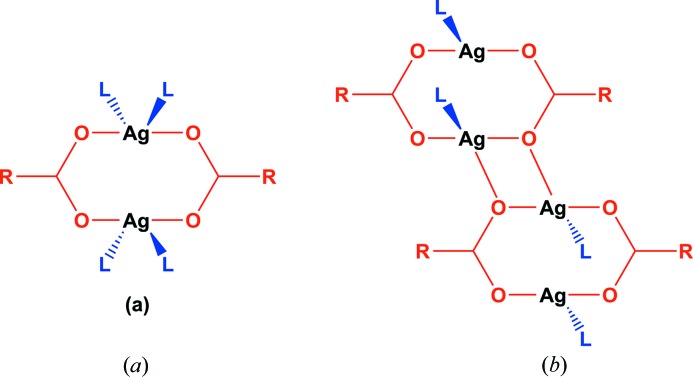
(*a*) Silver(I) carboxylate dimer and (*b*) silver(I) carboxylate tetramer secondary building units, which when connected by neutral ditopic ligands, L (here phenazine), propagate coordination polymers.

In recent years, our own work has examined solid–gas and solid–vapour reactions involving molecular crystals of coordination compounds that reversibly react with HCl and HBr gases (Mínguez Espallargas *et al.*, 2006 ▶, 2007 ▶, 2010 ▶, 2011 ▶; Vitórica-Yrezábal *et al.*, 2011 ▶), and coordination polymers that reversibly take up and release small alcohol molecules (Libri *et al.*, 2008 ▶; Vitórica-Yrezábal *et al.*, 2013 ▶), in each case requiring changes in metal coordination environments that are accompanied by structural changes and changes in intermolecular interactions (hydrogen bonding and/or halogen bonding). The present study builds upon earlier work that introduced a variety of networks formed by combining silver(I) perfluorocarboxylates with neutral ditopic ligands such as pyrazines and in particular emphasized the silver carboxylate dimer, Ag_2_(O_2_C*R*)_2_, as a secondary building unit (SBU) that can be linked by ditopic ligands into coordination polymers (Fig. 1 ▶). Specifically we report a new family of coordination polymers that involve tetrameric units Ag_4_(O_2_C*R*)_4_, which arise from fusing of two dimers *via* additional Ag—O bonds. The tetramers are linked *via* phenazine ligands to form coordination polymers, and exhibit a variety of chemical and structural transformations that result from loss of arene guests that directly coordinate to Ag(I) centres. This behaviour is related to earlier studies of a family of coordination polymers that comprise tetramethylpyrazine linker ligands (Libri *et al.*, 2008 ▶; Vitórica-Yrezábal *et al.*, 2013 ▶) rather than phenazine, but here we also report on the transformation between coordination polymer structures, both by heating and vapour-assisted means, in one case leading to a polymorph that is inaccessible in a phase-pure form through direct solution-phase synthesis.

## 2. Experimental   

### 2.1. Crystal syntheses   

All starting materials were purchased from Aldrich, Alfa Aesar or Fluorochem and used as received. Light was excluded from all reactions using foil to minimize decomposition to silver metal. In each case, 0.05 *M* solutions of the reagents were separately prepared by dissolving silver(I) heptafluorobutanoate (128 mg, 0.400 mmol) or phenazine (72 mg, 0.400 mmol) in 8 ml of solvent. In all cases, large yellow crystals suitable for single-crystal X-ray diffraction were formed within 1 week.

#### 2.1.1. [Ag_4_(O_2_C(CF_2_)_2_CF_3_)_4_(phen)_2_(tol)]·2(tol), **1-tol·tol**   

An 0.05 *M* solution of Ag(O_2_C(CF_2_)_2_CF_3_) (128 mg, 0.400 mmol) in 8 ml methanol was layered onto a 0.05 *M* solution of phenazine (72 mg, 0.400 mmol) in 8 ml toluene. Yield 71% (135 mg, 0.071 mmol). Anal. found: C 37.41, H 1.75, N 2.90; calcd: C 38.15, H 2.01, N 2.92%. Samples allowed to air-dry for more than 10 min: found: C 29.16, H 0.70, N 3.10; calcd. (for [Ag_4_(O_2_C(CF_2_)_2_CF_3_)_4_(phen)_2_], **2*b***): C 29.22, H 0.98, N 3.41%. Synthesis was also possible if methanol was replaced by ethanol, *n*-propanol or 2-propanol.

#### 2.1.2. [Ag_4_(O_2_C(CF_2_)_2_CF_3_)_4_(phenazine)_2_(p-xylene)_2_], **1-pxyl**   

A 0.05 *M* solution of Ag(O_2_C(CF_2_)_2_CF_3_) (128 mg, 0.400 mmol) in 8 ml methanol was layered onto a 0.05 *M* solution of phenazine (72 mg, 0.400 mmol) in 8 ml *p*-xylene. Yield 29.6% (55 mg, 0.030 mmol). Anal. found: C 36.24, H 1.70, N 2.51; calcd: C 36.23, H 1.95, N 3.02%. Samples heated at 120°C for 2 h: found: C 29.23, H 0.74, N 3.37; calcd (for [Ag_4_(O_2_C(CF_2_)_2_CF_3_)_4_(phen)_2_], **2*a*** or **2*b***): C 29.22, H 0.98, N 3.41%.

#### 2.1.3. [Ag_4_(O_2_C(CF_2_)_2_CF_3_)_4_(phenazine)_2_(*m*-xylene)_2_], **1-mxyl**   

An 0.05 *M* solution of Ag(O_2_C(CF_2_)_2_CF_3_) (128 mg, 0.400 mmol) in 8 ml methanol was layered onto a 0.05 *M* solution of phenazine (72 mg, 0.400 mmol) in 8 ml *m*-xylene. Yield 37.7% (70 mg, 0.038 mmol). Anal. found: C 36.28, H 1.57, N 2.61%; calcd: C 36.23, H 1.95, N 3.02%. Samples heated at 120°C for 2 h: found: C 29.46, H 0.90, N 3.44; calcd: C 29.22, H 0.98, N 3.41% (for [Ag_4_(O_2_C(CF_2_)_2_CF_3_)_4_(phen)_2_], **2*a*** or **2*b***). (See the supporting information for a discussion of X-ray powder diffraction and TGA.)

#### 2.1.4. [Ag_4_(O_2_C(CF_2_)_2_CF_3_)_4_(phenazine)_2_(tol)_x_((*p*-xylene)_1 − *x*_]·*n*(toluene)·(2 − *n*)(*p*-xylene), **1-pxyl-tol·pxyl·tol**   

A 0.05 *M* solution of Ag(O_2_C(CF_2_)_2_CF_3_) (128 mg, 0.400 mmol) in 8 ml methanol was layered onto a 0.05 *M* solution of phenazine (72 mg, 0.400 mmol) in 8 ml of 1:1 toluene:*p*-xylene. Yield 56.8% (110 mg, 0.057 mmol). Anal. found: C 37.96, H 1.98, N 2.74; calcd: C 38.67, H 2.22, N 2.90% (for *x* = 0.575, *n* = 2*x*; as found by GC (gas chromatography)/NMR).

#### 2.1.5. [Ag_4_(O_2_C(CF_2_)_2_CF_3_)_4_(phenazine)_2_], **2*a***   

A 0.05 *M* solution of Ag(O_2_C(CF_2_)_2_CF_3_) (128 mg, 0.400 mmol) in 8 ml methanol was layered onto a 0.05 *M* solution of phenazine (72 mg, 0.4 mmol) in 8 ml of *o*-xylene. Yield 45.6% (75 mg, 0.046 mmol). Anal. found: C 29.32, H 0.49, N 3.27; calcd: C 29.22, H 0.98, N 3.41%. Synthesis was also possible in comparable yields by layering a 0.05 *M* solution of Ag(O_2_C(CF_2_)_2_CF_3_) (128 mg, 0.400 mmol) in 8 ml methanol onto a 0.05 *M* solution of phenazine (72 mg, 0.400 mmol) in 8 ml of dichloromethane. Synthesis could also be achieved by slowly evaporating an 0.05 *M* solution of Ag(O_2_C(CF_2_)_2_CF_3_) (128 mg, 0.400 mmol) and phenazine (72 mg, 0.40 mmol) in 16 ml of acetone.

### 2.2. Vapour exposure experiments   

In all cases, crystals were removed from the mother liquor and gently dried between filter papers, before being gently ground in an agate pestle and mortar. The powder (approx. 30 mg) was placed in a small sample vial with a plastic lid, pierced once. This vial was placed inside a larger vial containing 1 ml of the relevant solvent, and the larger vial sealed and stored in the dark for 2 weeks.

### 2.3. Mechanochemistry   

100 mg of the dried, yellow microcrystalline **2*a*** was ground gently in an agate pestle and mortar, then placed into a 5 ml capacity (No. 59) Retsch cylindrical stainless steel grinding jar, either dry or with 50 µL acetone. The jar was fixed into a Retsch MM200 mixer mill, and shaken at a rate of 25 Hz for 15 min. The resultant yellow powder was analysed by X-ray powder diffraction.

### 2.4. Analytical techniques   

#### 2.4.1. X-ray crystallography   

Single-crystal X-ray data were collected at 100 K for compounds **1-tol·tol**, **1-mxyl**, **1-pxyl**, **1-tolpxyl** and **2*a*** on Bruker APEX-II diffractometers, using Mo *K*α radiation. Data for **2*b***, the product of heating **1·tol·tol**, were collected on a Rigaku Saturn 724+ CCD diffractometer at Diamond Light Source beamline I19 [λ = 0.6889 (3) Å] (Nowell *et al.*, 2012 ▶). Data were collected as a series of five sequences of frames, each covering approximately one hemisphere of reciprocal space. The first 20 frames of the first sequence were repeated at the end of data collection as a check for radiation damage. Each frame was collected as a 1 s exposure, with full available attenuation to prevent beam damage. CCD frame data were transformed from Rigaku to Bruker *SMART* format using the program *ECLIPSE* (Dawson *et al.*, 2004 ▶). Data were corrected for absorption using empirical methods (*SADABS*), based on symmetry-equivalent reflections combined with measurements at different azimuthal angles (Sheldrick, 1995 ▶; Blessing, 1995 ▶). Crystal structures were solved and refined against all *F* ^2^ values, using the *SHELXTL* program suite (Sheldrick, 2008 ▶), or using *Olex2* (Dolomanov *et al.*, 2009 ▶). Non-H atoms were refined anisotropically (except as noted), and H atoms placed in calculated positions refined using idealized geometries (riding model) and assigned fixed isotropic displacement parameters. Disorder in the fluoroalkyl chains in compounds **1-tol·tol**, **1-mxyl**, **1-tolpxyl** and **2*a*** was modelled in two orientations, dependent upon rotation about the β- and γ-CF_2_ groups and the terminal CF_3_ groups. Disordered atoms in the fluoroalkyl chains were modelled isotropically. Toluene molecules in the compound **1-tol·tol** are situated on inversion centres that lie at the centre of the six-membered ring. For **1-tol-pxyl·tol·pxyl** occupational and positional disorder for toluene/*p*-xylene guest molecules was dealt with by applying bond distance restraints and assigning fixed occupancies of 0.7215 to methyl C atoms consistent with the spectroscopic/chromatographic determination of the toluene:*p*-xylene ratio as 0.575:0.425. The non-coordinated arenes were modelled with isotropic displacement parameters. Crystallographic data for all compounds are summarized in Table 1 ▶.

#### 2.4.2. Powder X-ray diffraction   

Samples prepared as described above were loaded into borosilicate capillaries of diameter 0.7 mm (Diamond Light Source and ESRF) or 0.5 mm (University of Sheffield). For *in situ* heating studies, a small plug of glass wool was added to prevent sample loss from open capillaries during sample spinning. Data were collected on beamline I11 (Thompson *et al.*, 2009 ▶, 2011 ▶), at Diamond Light Source for the *in situ* heating study for **1·tol·tol** [λ = 0.826008 (2) Å], for compounds **1-tol-pxyl·tol·pxyl**, **1·mxyl**, **1·pxyl** and **2*a*** and for the *in situ* heating studies for **1·mxyl** and **1·pxyl** [λ = 0.826136 (2) Å], and for the *ex situ* alcohol vapour exposure study on **1·tol·tol** [λ = 0.82562 (1) Å]. Data were collected using a wide-angle (90°) PSD (position-sensitive detector) (Thompson *et al.*, 2011 ▶) comprised of 18 Mythen-2 modules. Each 2 s scan was collected as two 1 s scans with a 0.25° 2θ offset (to account for the gaps between the Mythen-2 modules). These pairs of scans were then summed to give a single data file, used for fitting.

Diffraction data for the products of solvent-assisted transformations of **2*b*** to mixtures of **2*b*** and **2*a***, as well as for the products of attempted syntheses of **2*b*** were collected on a Stoe Stadi P diffractometer using Cu *K*α radiation (λ = 1.5406 Å) in the Department of Materials Science and Engineering, University of Sheffield. Data were collected using a PSD detector with a single scan (5 < 2θ < 40°) at a scan rate of 0.067° min^−1^, using a rotating capillary.

Diffraction data for samples of **1-tol·tol** exposed to xylene vapours were collected at room temperature at λ = 0.3997939 (15) Å; and for the products of mechanochemical experiments on **2*a*** at 0.400021 (9) Å using station ID31^3^ (Fitch, 2004 ▶), at the European Synchrotron Radiation Facility (ESRF). The data were collected using a nine-channel multi-analyser crystal (MAC) detector. Using a rotating capillary, 5 scans (−2.5 ≤ 2θ ≤ 18 °) were collected at a speed of 4° min^−1^. After each scan the capillary was translated such that each scan was on a portion of sample not thus far irradiated. All patterns were summed to give a final pattern used for the data analysis.

Diffraction patterns were indexed and fitted using the *TOPAS-Academic* program (Coelho, 2007 ▶), by Pawley refinement (Pawley, 1981 ▶) for data with *d*_min_ ≤ 1.55 Å in each case, and (in cases specified) then by Rietveld refinement (Rietveld, 1969 ▶) using starting models from previous single-crystal structure determinations. Full details of refinements and all fitted patterns are included in the supporting information.

#### 2.4.3. Elemental analysis   

Elemental analyses were carried out by the University of Sheffield Department of Chemistry elemental analysis service, using a Perkin–Elmer 2400 CHNS/O Series II elemental analyser. Elemental analyses on the **1-arene** series of compounds were conducted immediately upon removal of the crystals from the mother liquor; measurements repeated after 30 min refrigeration at 5°C gave consistent values.

#### 2.4.4. ^1^H NMR spectroscopy   

A sample of **1-tol-pxyl·tol·pxyl** was air-dried and dissolved in DMSO-d_6_, then filtered through cotton wool. A ^1^H NMR spectrum was measured on a Bruker AV 400 MHz spectrometer. The spectrum is reported in the supporting information.

#### 2.4.5. Gas chromatography   

Crystals of **1-tol-pxyl·tol·pxyl** were dissolved in DMSO with some sonication, sealed in glass vials using crimped caps, and then run through a Perkin–Elmer Autosystem FID microcolumn, heating from 50 to 300°C at 10°C min^−1^. Retention times were compared to those for pure samples of phenazine, silver(I) heptafluorobutanoate, toluene, xylene (each dissolved in or diluted with DMSO) and pure DMSO. Relative content of guests was determined by direct comparison of chromatogram peak areas. The gas chromatogram for **1-tol-pxyl·tol·pxyl** can be found in the supporting information.

#### 2.4.6. Thermal analysis   

Thermogravimetric analyses were conducted using a Perkin–Elmer Pyris1 TGA model thermogravimetric analyser. Samples were heated from 30 to 400°C at 5°C min^−1^ under a flow of dry N_2_ gas. Thermogravimetric traces can be found in the supporting information.

## 3. Results and discussion   

### 3.1. Synthesis and crystal structures of coordination polymers 1-tol·tol, 1-pxyl, 1-mxyl, 1-tol-pxyl·tol·pxyl, **2*a*** and **2*b***   

A family of one-dimensional coordination polymers [Ag_4_(O_2_C(CF_2_)_2_CF_3_)_4_(phenazine)_2_(toluene)]·2(toluene) (**1-tol·tol**), [Ag_4_(O_2_C(CF_2_)_2_CF_3_)_4_(phenazine)_2_(*m*-xylene)_2_] (**1-mxyl**) and [Ag_4_(O_2_C(CF_2_)_2_CF_3_)_4_(phenazine)_2_(*p*-xylene)_2_] (**1-pxyl**) were synthesized by layering a methanol solution of silver heptafluorobutanoate onto a solution of phenazine in the corresponding arene. All compounds were characterized by single-crystal X-ray diffraction, the composition was confirmed by elemental analysis and phase purity was examined by X-ray powder diffraction (see the supporting information). The mixed-arene coordination polymer [Ag_4_(O_2_C(CF_2_)_2_CF_3_)_4_(phenazine)_2_(toluene)_*n*_(*p*-xylene)_1 − *n*_]·*n*(toluene)·(2 − *n*)(*p*-xylene) (**1-tol-pxyl·tol·pxyl**) was prepared in an analogous manner using a 1:1 toluene:*p*-xylene solvent mixture. When *o*-xylene is used as the arene, the product is one-dimensional coordination polymer [Ag_4_(O_2_C(CF_2_)_2_CF_3_)_4_(phenazine)_2_] (**2*a***), which contains no *o*-xylene. A two-dimensional coordination polymer, **2*b***, which is a polymorph of **2*a***, is obtained upon loss of toluene from **1-tol·tol**, but could not be prepared from solution-phase synthesis.

**Figure 2:**
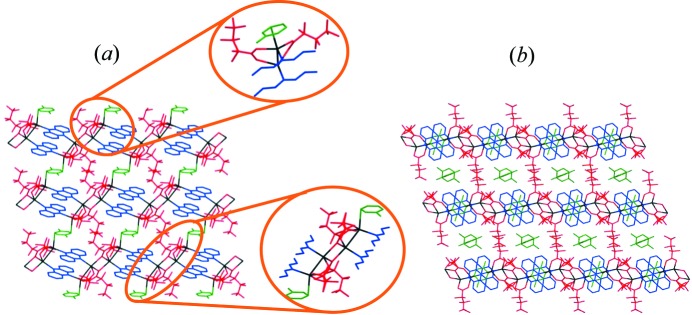
Crystal structure of [Ag_4_(O_2_C(CF_2_)_2_CF_3_)_4_(phenazine)_2_(toluene)]·2(toluene) (**1-tol·tol**) shown (*a*) in the *ab* plane, highlighting the μ:η^1^,η^1^ bridging toluene molecules (expansions showing the silver carboxylate dimer and the tetramers; only one of the two toluene orientations is shown in the expansions) and (*b*) in the *bc* plane, highlighting the non-coordinated toluene solvent between two-dimensional layers of **1-tol·tol**. Silver ions shown in black, heptafluorobutanoate in red, phenazine in blue and toluene in green. H atoms omitted for clarity.

The crystal structure of **1-tol·tol** (Fig. 2 ▶) consists of building blocks of Ag_4_(O_2_C(CF_2_)_2_CF_3_)_4_(phenazine)_2_, within which two Ag_2_(O_2_C(CF_2_)_2_CF_3_)_2_ dimers are linked by bridging phenazine ligands which are oriented in a face-to-face manner. These building blocks are linked into one-dimensional tapes *via* pairs of Ag—O bonds, forming the silver(I) carboxylate tetramer illustrated in Fig. 1 ▶(*b*). The one-dimensional tapes are cross-linked *via* toluene molecules, which bridge silver(I) centres in a μ–η^1^,η^1^ manner. Although there are many examples of π-coordination to Ag(I) centres, to the best of our knowledge, this toluene bridging mode has not previously been observed, although bridging in a μ–η^2^,η^2^ fashion has been reported (Zhong *et al.*, 2001 ▶). The cross-linking creates two-dimensional layers of silver coordination polymer, with an additional two toluene molecules per formula unit residing between the layers (Fig. 2 ▶ *b*).

**Figure 3:**
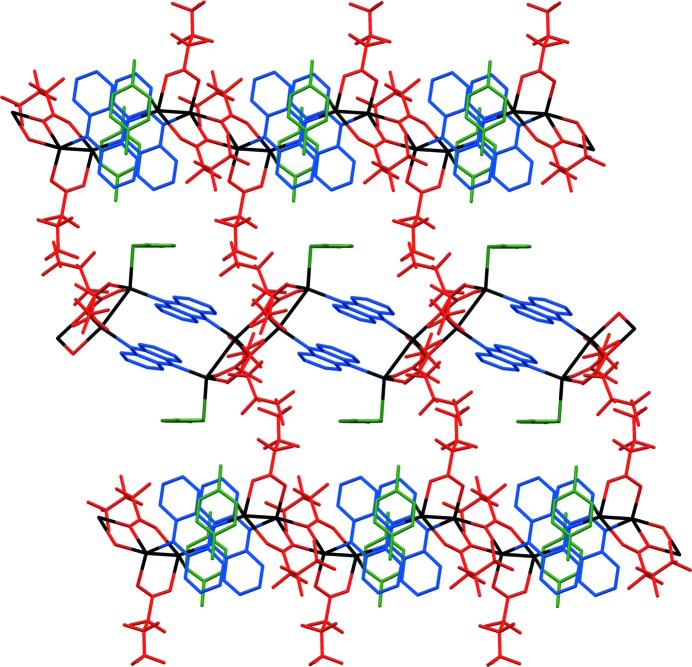
Stacking of one-dimensional coordination polymer chains in **1-pxyl**, [Ag_4_(O_2_C(CF_2_)_2_CF_3_)_4_(phenazine)_2_(*p*-xylene)_2_]. H atoms omitted for clarity. Colours are as in Fig. 1 ▶. **1-mxyl** is isostructural with **1-pxyl**.

Although synthesis conditions using *p*-xylene or *m*-xylene instead of toluene are otherwise unchanged, the coordination polymers generated, **1-pxyl** and **1-mxyl**, are similar, but not identical to **1-tol·tol**. Each comprises the same polymer tape structure as **1-tol·tol**, except that the coordinated *m*-xylene or *p*-xylene does not bridge Ag(I) centres, instead coordinating in a η^1^ fashion only to the outer Ag(I) centres of each silver carboxylate tetramer (Fig. 3 ▶). These one-dimensional polymer tapes stack such that there are also no additional guest solvent molecules between polymers. Alternate one-dimensional coordination polymer tapes are mutually rotated by 90° along the polymer axis, such that the orientation of the phenazine ligands is orthogonal in adjacent tapes, which also facilitates edge-to-face C—H⋯π interactions between phenazine ligands and xylenes.

Combining silver heptafluorobutanoate and phenazine in a methanol/1:1 toluene:*p*-xylene solvent system yielded coordination polymer **1-tol-pxyl·tol·pxyl**, [Ag_4_(O_2_C(CF_2_)_2_CF_3_)_4_(phenazine)_2_(toluene)_*n*_(*p*-xylene)_1 − *n*_]·2*n*(toluene)·(2 − 2*n*)(*p*-xylene), which is isostructural with **1-tol·tol**. The proportion of the two arene guests within the crystal at the coordinated and uncoordinated sites could not be determined reliably from the single-crystal diffraction experiment. However, no clear difference between the populations of the two sites was apparent. The relative proportions of the two arenes were analysed, upon dissolution of the coordination polymer, by ^1^H NMR spectroscopy (integration of signals) and by gas chromatography (peak areas), and toluene:*p*-xylene ratios of 58:42 and 56:44, respectively, were determined. This suggests at best only a slight preference for toluene inclusion over *p*-xylene inclusion into **1-tol-pxyl·tol·pxyl**. The different crystal structures of **1-tol·tol** and **1-pxyl** suggest that toluene rather than *p*-xylene should be present in the bridging coordination sites, but this could not be established crystallographically. In the final model for the crystal structure the populations of all arene sites were assumed to be identical with a toluene:*p*-xylene ratio of 57.5:42.5 (*i.e.* *n* = 0.575).

**Figure 4:**
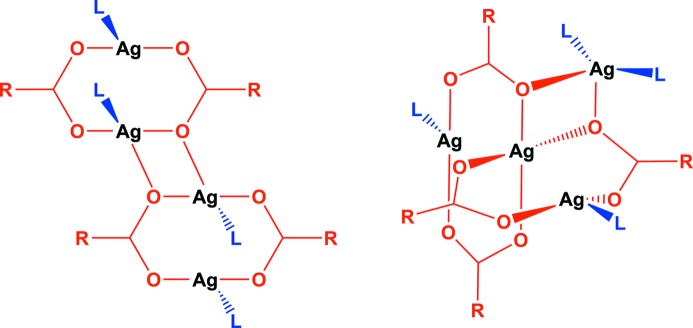
The silver(I) carboxylate tetramers propagating coordination polymers **1-tol·tol**, **1-pxyl** and **1-mxyl** (left) and those in polymer architecture **2*a*** (right).

**Figure 5:**
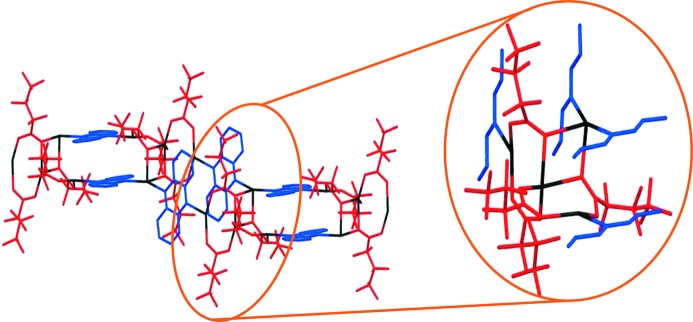
Coordination polymer **2*a***, showing the coordination environment around Ag(I) centres; the expansion has been rotated for clarity. H atoms are omitted for clarity. Colours are as in Fig. 1 ▶.

Conducting the coordination polymer synthesis using a methanol/*o*-xylene solvent system yielded the one-dimensional coordination polymer **2*a***, [Ag_4_(O_2_C(CF_2_)_2_CF_3_)_4_(phenazine)_2_]; no arene solvent is included in the crystal structure, in contrast to the use of other xylenes or toluene. Like coordination polymers **1-tol·tol**, **1-pxyl** and **1-myxl**, the structure of **2*a*** consists of Ag_4_(O_2_C(CF_2_)_2_CF_3_)_4_ tetramers linked by pairs of parallel phenazine units that bridge between Ag(I) centres to give a one-dimensional coordination polymer tape assembly. However, the tetramer units have a different configuration to those noted previously (Fig. 4 ▶), wherein one Ag(I) centre is exclusively coordinated by carboxylate ligands and another forms bonds to two phenazine ligands, whereas all four Ag(I) centres are each bonded to one phenazine ligand in **1-tol·tol**, **1-pxyl** and **1-myxl**. The coordination of phenazine ligands to the tetramer unit in **2*a*** leads to an arrangement in which alternate pairs of phenazine ligands within one tape are oriented orthogonally rather than parallel to other pairs (Fig. 5 ▶).

**Figure 6:**
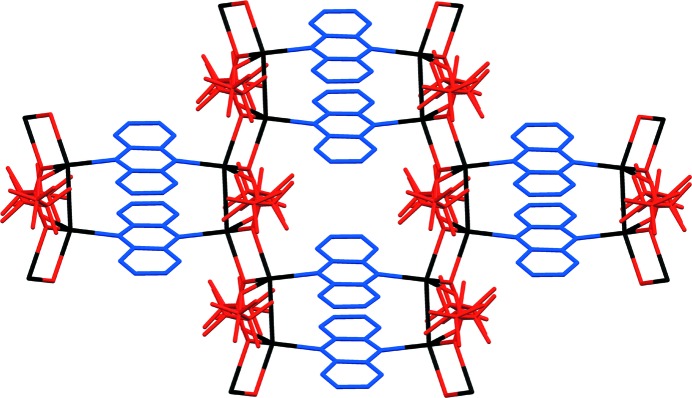
Coordination polymer **2*b***, viewed perpendicular to the layer arrangement. H atoms are omitted for clarity. Colours are as in Fig. 1 ▶.

A very small single crystal of the coordination polymer **2*b***, [Ag_4_(O_2_C(CF_2_)_2_CF_3_)_4_(phenazine)_2_], a polymorph of **2*a***, was recovered as a small fragment after heating crystals of **1-tol·tol** to remove toluene. Data collection at beamline I19 at Diamond Light Source enabled crystal structure determination. Unlike **2*a*** the structure of **2*b*** is a two-dimensional coordination polymer comprised of Ag_4_(O_2_C(CF_2_)_2_CF_3_)_4_(phenazine)_2_ units linked *via* additional Ag—O bonds (Fig. 6 ▶), analogous to that of the previously reported [Ag_4_(O_2_C(CF_2_)_2_CF_3_)_4_(TMP)_2_] (TMP = tetramethylpyrazine) (Vitórica-Yrezábal *et al.*, 2013 ▶).

### 3.2. Thermal, mechanochemical and vapour-assisted structural transformations   

**Figure 7:**
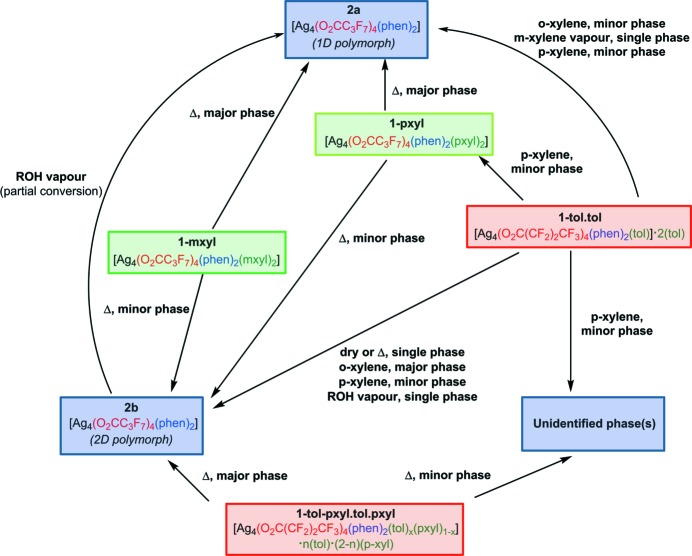
Structural conversions for coordination polymers **1**, **2*a*** and **2*b***. Exposure of crystals to solvent vapour is indicated by the name of the solvent; heating is indicated by ‘Δ’. Where mixtures of products resulted rather than conversion to a single phase, the major/minor phases are noted. (The reversibility of toluene loss by **1-tol·tol** was also examined by exposure of **2*b*** to toluene vapour. No reaction was observed.)

Thermogravimetric analysis of **1-tol·tol** and **1-pxyl** indicated facile loss of the arene to give materials of the composition of **2*a*** and **2*b*** (Figs. S2 and S3), as also confirmed by elemental analysis. Loss of toluene occurs most readily and is complete after 10 min in air at room temperature. Loss of *p*-xylene was from **1-pxyl** complete by 120°C in the TGA (thermogravimetric analysis) experiment, and heating **1-pxyl** or **1-mxyl** to 120°C for 2 h was shown to be sufficient to remove all xylene with no further losses. These observations led us to investigate further the chemical and structural changes occurring in the arene-loss processes, as well as to investigate their reversal upon exposure to arene vapours and more generally the behaviour of these materials upon exposure to arene or alcohol solvent vapours, and upon grinding, particularly in light of prior results involving reversible (alcohol) vapour uptake and structural conversions by the related ID coordination polymer system [Ag_4_(O_2_C(CF_2_)_2_CF_3_)_4_(TMP)_3_] (Libri *et al.*, 2008 ▶; Vitórica-Yrezábal *et al.*, 2013 ▶). A summary of the transformations identified is provided in Fig. 7 ▶.

#### 3.2.1. Thermal transformations   

**Figure 8:**
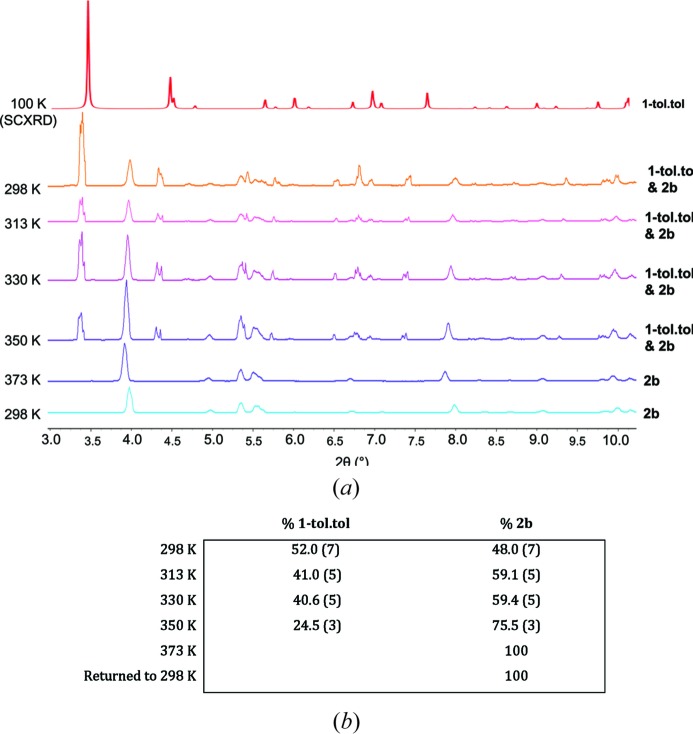
(*a*) *In situ* X-ray powder diffraction heating study showing the conversion of **1-tol·tol** to **2*b***. Patterns were measured at intervals of approximately 20 min over a period of 2 h. The top pattern is calculated from single-crystal structure determination of **1-tol·tol**. (*b*) Relative quantities of the two phases present determined by Rietveld refinement (see also Figs. S6–S11).

Heating a powdered crystalline sample in a capillary during an *in situ* synchrotron X-ray powder diffraction experiment (Fig. 8 ▶) enabled the loss of toluene from **1-tol·tol** to be followed and confirmed the sole product to be two-dimensional coordination polymer **2*b***. Conversion of **1-tol·tol** to **2*b*** proceeds directly without any detected crystalline intermediate phases. Rietveld analysis indicated that the starting material had already undergone some toluene loss and conversion to **2*b*** (48%) and indicated full conversion after approximately 100 min of heating, by which time the temperature had been increased to 373 K.

Solid-state conversion of **1-tol·tol** to **2*b*** involves breaking of Ag—π(toluene) bonds and the loss of all toluene coupled with the formation of new Ag—O bonds to form the more condensed material **2*b***. The process resembles that previously observed for conversion of a one-dimensional coordination polymer [Ag_4_(O_2_C(CF_2_)_2_CF_3_)_4_(TMP)_3_] to a two-dimensional layered coordination polymer [Ag_4_(O_2_C(CF_2_)_2_CF_3_)_4_(TMP)_2_] through loss of ligand TMP, which bridges between Ag_4_(O_2_C(CF_2_)_2_CF_3_)_4_(TMP)_2_ units, and the formation of new Ag—O bonds between the Ag_4_(O_2_C(CF_2_)_2_CF_3_)_4_(TMP)_2_ units (Vitórica-Yrezábal *et al.*, 2013 ▶). In this previous case the transformation was found to be irreversible, and indeed exposure of **2*b*** to toluene vapour did not result in conversion back to **1-tol·tol**, but instead left the material unchanged. It is worth noting that phase-pure **2b** could only be accessed through heating/solvent loss from **1-tol·tol**. Arene loss from **1-tol-pxyl·tol·pxyl** was examined in an *ex situ* diffraction study. A powder sample was allowed to air-dry for 30 min. Analysis by synchrotron X-ray powder diffraction confirmed partial transformation to **2*b***, but also the presence of an unidentified phase (Fig. S1).

**Figure 9:**
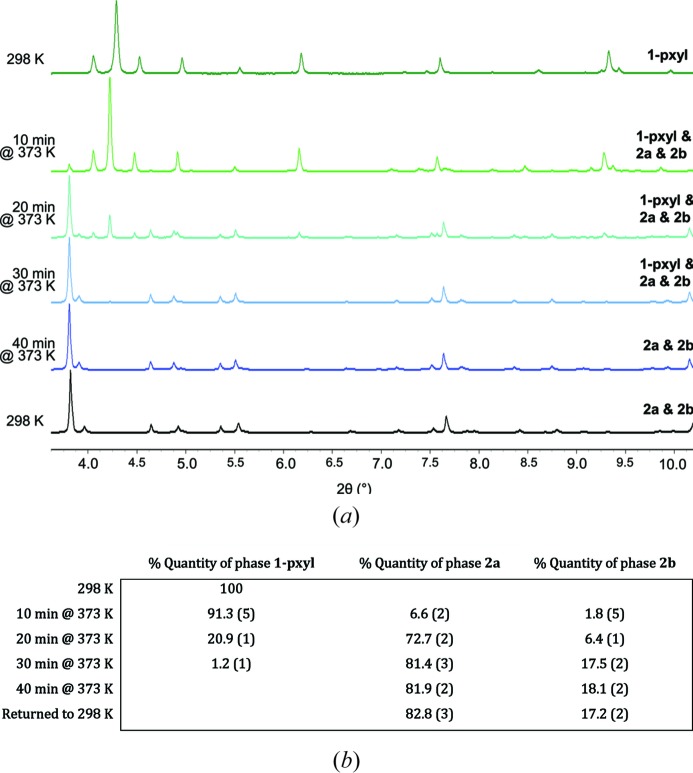
(*a*) *In situ* X-ray powder diffraction heating study showing conversion of **1-pxyl** to a mixture of **2*a*** and **2*b***. (*b*) Relative quantities of the two phases present determined by Rietveld refinement (see also Figs. S12–S17).

*In situ* X-ray powder diffraction heating studies were also conducted on **1-pxyl** (Fig. 9 ▶) and **1-mxyl** (Fig. S18). The samples were heated to 373 K while being monitored by powder diffraction. For **1-pyxl**, Pawley and Rietveld fitting of the powder diffraction patterns indicated the formation of both polymorphs **2*a*** and **2*b*** as a result of loss of *p*-xylene, with polymorph **2*a*** as the major product. Upon complete loss of *p*-xylene, after 40 min, only **2*a*** and **2*b*** are present, consistent with earlier TGA and elemental analysis results. Rietveld analysis indicated that **2*a*** and **2*b*** are present in the ratio 82:18 in the final product. For **1-mxyl**, the starting material used was found to be already a mixture of **1-mxyl**, **2*a*** and **2*b***, suggesting some *m*-xylene loss prior to the initial diffraction measurement. Heating to 373 K resulted in complete conversion to a mixture of **2*a*** and **2*b*** after 4 min, with **2*a*** again as the major phase, although data quality was sufficient only for Pawley fitting and therefore did not enable quantitative analysis of composition.

**Figure 10:**
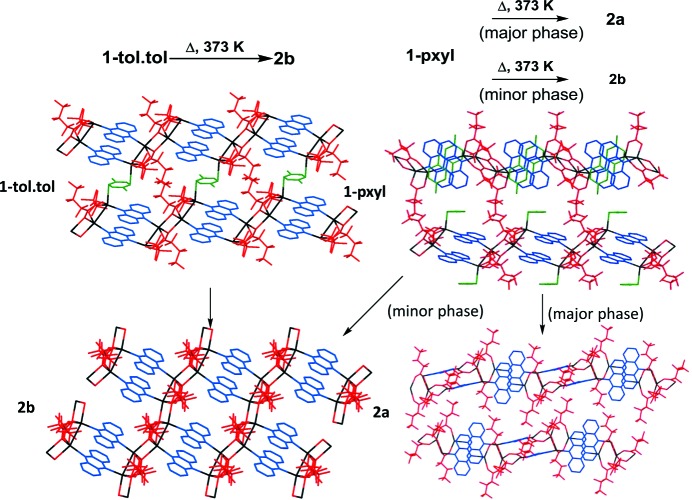
A comparison of the products of heating coordination polymers **1-tol·tol** and **1-pxyl**, suggesting the role of pre-organization on the structure of the products.

The thermal transformation of **1-pxyl** (majority phase) and **1-mxyl** to a one-dimensional coordination polymer architecture (coordination polymer **2*a***), consisting of orthogonally packed chains, similar to that of **1-pxyl** contrasts with the sole product of heating of **1-tol·tol**. The heating of **1-tol·tol**, a two-dimensional coordination polymer propagated in one dimension by fused silver-carboxylate tetramers, pre-arranged such that they are co-planar, gives a like product, **2*b***. This indicates the role of pre-organization of the polymer chains on the polymorph products given, and prompted further investigation on potential inter-conversion between polymorph architectures **2*a*** and **2*b*** (Fig. 10 ▶).

#### 3.2.2. Vapour-assisted and mechanochemical transformations   

Crystals of **1-tol·tol** were dried and exposed to solvent vapour in attempts to facilitate exchange between arene guests, or the inclusion of alcohols in the silver carboxylate tetramer. These experiments were followed up (*ex situ*) by X-ray powder diffraction analysis to assess any phase changes.

Crystals of **1-tol·tol** exposed to *o*-xylene vapours gave mixed-phase products of polymorphs **2*a*** and **2*b***. When exposed to *m*-xylene, **1-tol·tol** lost toluene and was converted to phase **2*a***, or when exposed to *p*-xylene, Pawley fitting of the resultant powder pattern indicated the presence of **2*a*** and **2*b***, along with peaks consistent with **1-pxyl** and a further unidentified phase (Fig. S28). These vapour-assisted transformations to give the two polymorphs **2*a*** and **2*b*** prompted an investigation into their interconversion. Mechanochemical conversion of **2*a*** into **2*b*** by dry grinding (Fig. S10) and by liquid-assisted grinding (LAG) using acetone (Fig. S11) was unsuccessful. Similarly, exposure of **2*a*** to toluene vapour resulted in no change in composition. However, exposure of crystals of **2*b*** to alcohol vapour (methanol, ethanol or 2-propanol) for a period of 2 weeks did facilitate a partial transformation to **2*a***.^1^ X-ray powder diffraction analysis of these samples (*ex situ*) and Rietveld fitting indicated that after 2 weeks the material comprised approximately 60% two-dimensional polymorph **2*b*** and 40% one-dimensional polymorph **2*a***. The partial conversion of polymorph **2*b*** to polymorph **2*a*** may suggest that the conversion of **1-tol·tol** to **2*b*** by vapour-assisted means may continue on to polymorph **2*a***. However, a sample of **1-tol·tol** gave only phase-pure **2*b*** (Fig. S8) when exposed to ethanol vapour for 2 weeks. The tendency of vapour-assisted transformations of materials **1-tol·tol** and **2*b*** towards the one-dimensional polymorph **2*a*** may indicate that **2*a*** is thermodynamically the more stable material, but the measurements made are not able to provide full details of the mechanism.^2^

#### 3.2.3. Arene separation   

Separation of isomers of small molecules has been examined for a number of porous materials. Such separations have been demonstrated with zeolites (Bellat *et al.*, 1995 ▶), but discrimination between isomers in adsorption has also been reported for MOFs (Alaerts *et al.*, 2008 ▶; Gu & Yan, 2010 ▶; Bárcia *et al.*, 2011 ▶; El Osta *et al.*, 2012 ▶; Warren *et al.*, 2014 ▶) and crystalline clathrates (Lusi & Barbour, 2012 ▶, 2013 ▶). In the work reported here, it is clear that exposure to different arenes (toluene and xylenes) during synthesis of **1** leads to different structures being formed, depending on the preferred interactions of the arene with the coordination network. Furthermore, exposure to vapours of different xylene isomers leads to different solid-state transformations (*e.g.* for **1-tol.tol**), indicating that there is discrimination between different arenes. However, since these observed processes do not involve simple adsorption/desorption of the arenes, it is less likely that these materials could be used in their current form for effective separation of xylenes.

## 4. Conclusions   

We have reported a family of one-dimensional silver(I) perfluorocarboxylate coordination polymers constructed from {Ag_4_(O_2_C(CF_2_)_2_CF_3_)_4_(phenazine)_2_} building blocks linked through additional Ag–O bonds and containing η^1^-bound arenes, including an unusual μ:η^1^,η^1^-toluene ligand material **1-tol.tol**. All can lose the entrapped and coordinated arene molecules either at ambient temperature or upon mild heating to convert to either a one-dimensional or two-dimensional coordination polymer, **2a** and **2b**, respectively, which are polymorphs of composition Ag_4_(O_2_C(CF_2_)_2_CF_3_)_4_(phen­azine)_2_. These transformations have been followed by *in situ* X-ray powder diffraction and TGA. Further exploration of these transformations has been undertaken by examining the exposure of the parent materials and the products to different solvent vapours and in some instances to mechanochemical force. The results, also verified by X-ray powder diffraction, illustrate the potential to harness chemical and structural transformations in the solid state involving labile metal–ligand bonds.

## Supplementary Material

Crystal structure: contains datablock(s) 1-tol.tol, 1-pxyl, 1-mxyl, 2a, 2b, 1-tol-pxyl.tol.pxyl. DOI: 10.1107/S2052252515000147/bi5040sup1.cif

XRPD, NMR and TGA data. DOI: 10.1107/S2052252515000147/bi5040sup2.pdf

CCDC references: 1051373, 1051374, 1051375, 1051376, 1051377, 1051378

## Figures and Tables

**Table:** 

	**1-toltol**	**1-pxyl**	**1-mxyl**
Crystal habit	Plate	Plate	Plate
Crystal colour	Yellow	Yellow	Yellow
Crystal size (mm)	0.41 0.14 0.11	0.37 0.33 0.08	0.42 0.20 0.12
Crystal system	Triclinic	Monoclinic	Monoclinic
Space group	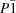	*P*2_1_/*c*	*P*2_1_/*c*
*a* ()	10.6531(7)	11.1146(3)	11.3750(6)
*b* ()	11.2628(7)	22.7107(8)	22.5963(10)
*c* ()	14.4311(10)	13.2046(4)	13.3460(7)
()	72.401(3)	90	90
()	86.598(3)	111.785(2)	113.472(2)
()	82.882(3)	90	90
*V* (^3^)	1637.3(2)	3095.1(2)	3146.5(3)
Density (mgm^3^)	1.948	1.992	1.959
Temperature (K)	100	100	100
_(Mo*K*)_ (mm^1^)	1.316	1.388	1.366
range ()	2.40327.116	2.6725.74	2.4527.43
No. of measured reflections	25299	26785	25586
No. of independent reflections, *R*_int_	7283, 0.0507	7075, 0.0505	7203, 0.0574
No. of reflections used in refinement, *n*	7283	7075	7203
LS parameters, *p*	422	453	432
Restraints, *r*	0	0	70
*R*1 (*F*)† *I* > 2.0(*I*)	0.0649	0.0407	0.0744
*wR*2 (*F^2^*)†, all data	0.1673	0.1217	0.1887
*S*(*F^2^*)†, all data	1.086	1.048	1.117

**Table:** 

	**2*a***	**2*b***	**1-tol-pxyltolpxyl**
Crystal habit	Plate	Plate	Plate
Crystal colour	Yellow	Yellow	Yellow
Crystal size (mm)	0.03 0.26 0.40	Not recorded	0.33 0.21 0.15
Crystal system	Monoclinic	Triclinic	Triclinic
Space group	*C*2/*c*	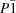	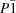
*a* ()	27.578(3)	10.782(3)	10.6658(15)
*b* ()	9.2670(10)	11.006(4)	11.2395(14)
*c* ()	21.211(2)	12.540(4)	14.325(2)
()	90	71.569(4)	72.054(2)
()	118.142(3)	76.089(4)	86.608(3)
()	90	62.229(4)	83.149(3)
*V* (^3^)	4779.9(9)	1241.5(7)	1621.6(4)
Density (mgm^3^)	2.285	2.199	1.985
Temperature (K)	100	100	100
_(Mo*K*)_ (mm^1^)	1.782	1.715	1.330
range ()	2.42024.224	2.23921.865	3.51824.387
No. of measured reflections	20167	9058	10048
No. of independent reflections, *R*_int_	5445, 0.0643	3916, 0.0357	7080, 0.0483
No. of reflections used in refinement, *n*	5445	3916	7080
LS parameters, *p*	362	315	382
Restraints, *r*	59	48	36
*R*1 (*F*)† *I* > 2.0(*I*)	0.0675	0.0804	0.0709
*wR*2 (*F^2^*)†, all data	0.2299	0.272	0.207
*S*(*F^2^*)†, all data	1.022	1.051	1.0196

† *R*1(*F*) = (|*F*_o_| |*F*_c_|)/|*F*_o_|; *wR*2(*F* ^2^) = [*w*(*F*_o_^2^ *F*_c_^2^)^2^/*wF*_o_^4^]^1/2^; *S*(*F* ^2^) = [*w*(*F*_o_^2^ *F*_c_^2^)^2^/(*n + r p*)]^1/2^.

## Footnotes

3 The beamline at ESRF was moved in 2014 from station ID31 to ID22.

1 Since **2*a*** can be formed in solution in the presence of methanol, without formation of **2*b***, it is possible that **2*b*** converts to **2*a*** by surface recrystallization in the presence of alcohols.

2 Mechanistic details are difficult to establish in the absence of *in situ* microscopy measurements (*e.g.* AFM), as illustrated by the findings of Khlobystov and co-workers (Thompson *et al.*, 2004 ▶; Cui *et al.*, 2009 ▶).
