# Supplementary material for: Solvent-vapour-assisted pathways and the role of pre-organization in solid-state transformations of coordination polymers
Source: IUCrJ. 2015 Feb 26;2(Pt 2):188–97. doi: 10.1107/S2052252515000147 (PMC4392413; doi:10.1107/S2052252515000147)
Supplement: Supplementary file 2 [file m-02-00188-sup2.pdf]

IUCrJ

Volume 2 (2015)

Supporting information for article:

Solvent vapour-assisted pathways and the role of pre-organisation in solid-state transformations of coordination polymers

James S. Wright, Iñigo J. Vitórica-Yrezábal, Harry Adams, Stephen P. Thompson, Adrian H. Hill and Lee Brammer

Supporting information

Solvent vapour-assisted pathways and the role of pre-organisation in solid-state transformations of coordination polymers

James S. Wright,[a] Iñigo J. Vitórica-Yrezábal,[a] Harry Adams,[a] Stephen P. Thomson,[b] Adrian H. Hill[c], and Lee Brammer*,[a]

[a] *Department of Chemistry, University of Sheffield, Brook Hill, Sheffield S3 7HF, UK. E-mail: lee.brammer@sheffield.ac.uk*

[b] *Diamond Light Source, Harwell Science and Innovation Campus, Didcot, Oxfordshire OX11 0DE, UK*

[c] *European Synchrotron Radiation Facility, 6 rue J. Horowitz, 38042 Grenoble, France. Present address: Johnson Matthey Technology Centre, Savannah, GA, USA.*

Table of Contents

1. Phase purity checks by XRPD — Page S2

2. Thermogravimetric analysis for **1-tol.tol**, **1-pxyl** and **1-mxyl** — Page S2

3. Gas chromatographic and NMR Spectroscopic analysis of **1-tol-pxyl.tol.pxyl** — Page S4

4. X-ray powder diffraction studies of heating **1-tol.tol**, **1-pxyl**, **1-mxyl** and **1-tol-pxyl.tol.pxyl** — Page S6

5. Phase identification by X-ray powder diffraction from *ex situ* vapour exposure experiments — Page S19

6. Phase identification by X-ray powder diffraction following grinding experiments — Page S24

7. References — Page S25

1. Phase-purity checks by XRPD

Although the composition of **1-tol.tol** was confirmed by elemental analysis, a phase-pure sample of **1-tol.tol** could not be studied by powder diffraction as toluene loss during sample loading resulted in formation of **2b** (see Figures 5 and S6).

Phase-purity of **1-pxyl** was confirmed by X-ray powder diffraction (see Figure S12).

Preparation of **1-mxyl** was inconsistent. Satisfactory elemental analysis were obtained to confirm composition. However, TGA measurements were inconsistent due to impurity phases. In situ heating studies of **1-mxyl** show impurities of **2a** and **2b** at the initial stage, but these could have been formed from loss of m-xylene as further heating induces loss of m-xylene and full conversion into a mixture of **2a** and **2b** (see Figure S18).

The composition of **1-tol-pxyl.tol.pxyl** was examined by elemental analysis as well as by NMR spectroscopy and gas chromatography upon dissolution, but rapid loss of arene guests prevented confirmation of phase-purity by X-ray powder diffraction.

Phase-purity of **2a** was established by PXRD (see Figure S1).

2b is synthesised by heating **1-tol.tol** and is formed in phase-pure form (see Figure S11).

Phase purity check, 2a. The yellow microcrystalline sample was loaded into a 0.7 mm borosilicate capillary. X-ray diffraction data were collected (λ = 0.826136(2) Å) at beamline I11 at Diamond Light Source,[S1,S2] equipped with a wide angle (90 °) PSD detector comprising 18 Mythen-2 modules. A pair of scans was conducted at room temperature, related by a 0.25 ° detector offset to account for gaps between detector modules. The resulting patterns were summed to give the final pattern for structural analysis. The powder pattern was indexed using the TOPAS program[S3] and unit cell dimensions were determined from a Pawley refinement,[S4] using 408 parameters (6 background, 1 zero error, 5 profile, 4 cell, 392 reflections), resulting in final indices of fit Rwp = 7.695, Rwp' = 19.123. The starting model used for the Rietveld refinement,[S6] conducted using TOPAS, was the single-crystal structure of **2a**, using 18 parameters (6 background, 1 zero error, 5 profile, 4 cell, 1 scale, 1 global scale factor for thermal parameters). Rietveld refinement converged to Rwp = 13.010, Rwp' = 31.066 (Figure S1). [a = 27.7574 (8) Å, b = 9.3715 (2) Å, c = 21.5624 (6) Å, β = 117.381 (3) °, V = 4980.6 (3) Å^3].

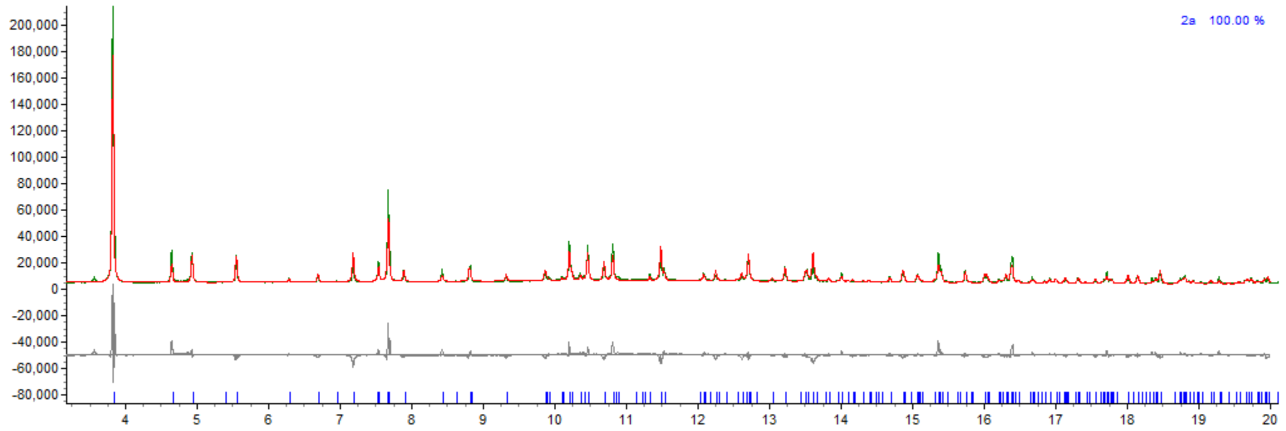

Figure S1. Observed (green) and calculated (red) profiles and difference plot [I_{obs}-I_{calc}] (grey) of the Rietveld refinement. (2θ range 3.0 – 20.0 °, d_{min} = 2.42 Å).

2. Thermogravimetric analysis for 1-tol.tol, 1-pxyl and 1-mxyl

Thermogravimetric analyses were conducted using a Perkin-Elmer Pyris1 TGA model thermogravimetric analyser. Samples were heated from 30 to 400 °C at 5 °C min^{-1}, under a nitrogen atmosphere.

[$Ag_4(O_2C(CF_2)_2CF_3)_4$(phenazine)$_2$(toluene)]·2(toluene), 1-tol.tol.

Mass loss begins immediately at room temperature or the first measurement temperature. Figure S2 shows a typical TGA trace. The initial mass loss in this measurement is 11.4%, but the value varied between repeat runs (range approx. 11-14%). This mass loss is consistent with the loss of toluene (expected mass loss 14.4%) assuming some toluene is lost prior to the start of measurements, and is consistent with the facile toluene loss observed during other measurements. A further mass loss, with onset at around 240 °C, consistently, corresponds to removal of remaining organic material silver(I) oxide (expected final mass 24.9%; observed final mass 23.8%).

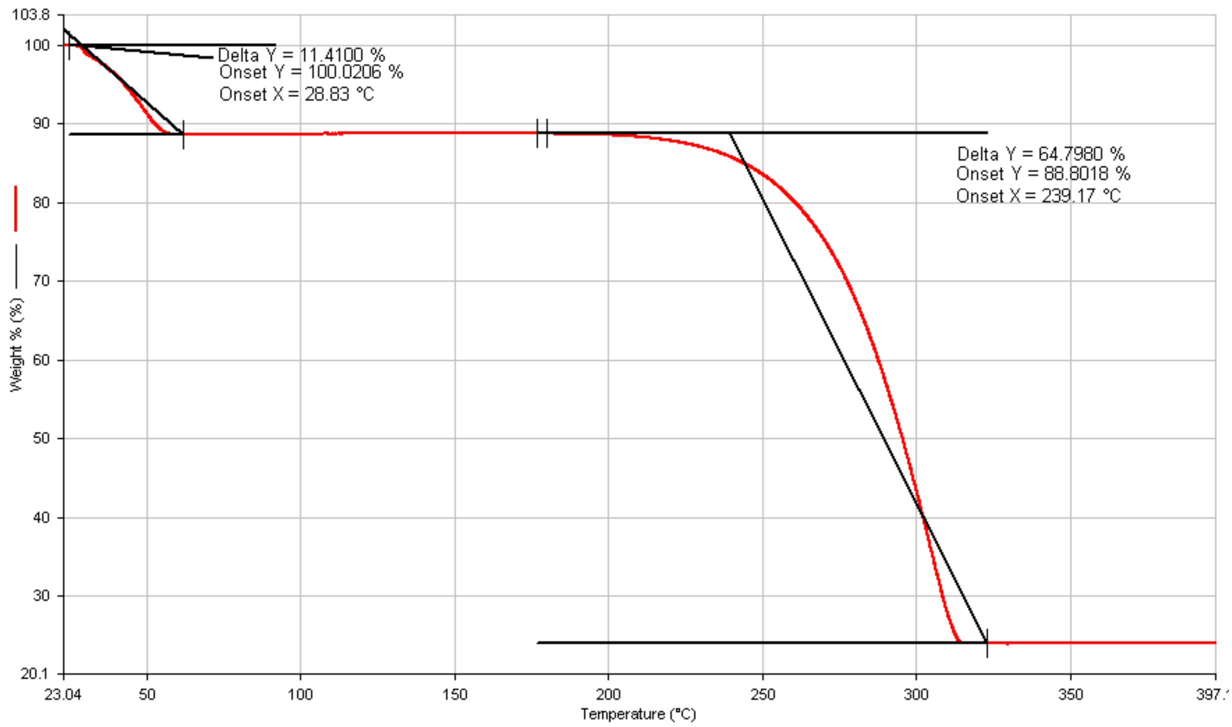

Figure S2. Thermogravimetric analysis trace and expected mass contribution table for **1-tol.tol**.

[$Ag_2(O_2C(CF_2)_2CF_3)_2$(phenazine)(*p*-xylene)], 1-pxyl.

The TGA trace for **1-pxyl** is shown in Figure S3. The initial observed mass loss of 11.4% (10.8/95.0) with onset at 74 °C corresponds well to loss of *p*-xylene (expected 11.5%). A further mass loss, with onset at around 280 °C consistently, corresponds to removal of remaining organic material silver(I) oxide (expected final mass 24.9%; observed final mass 23.4%).

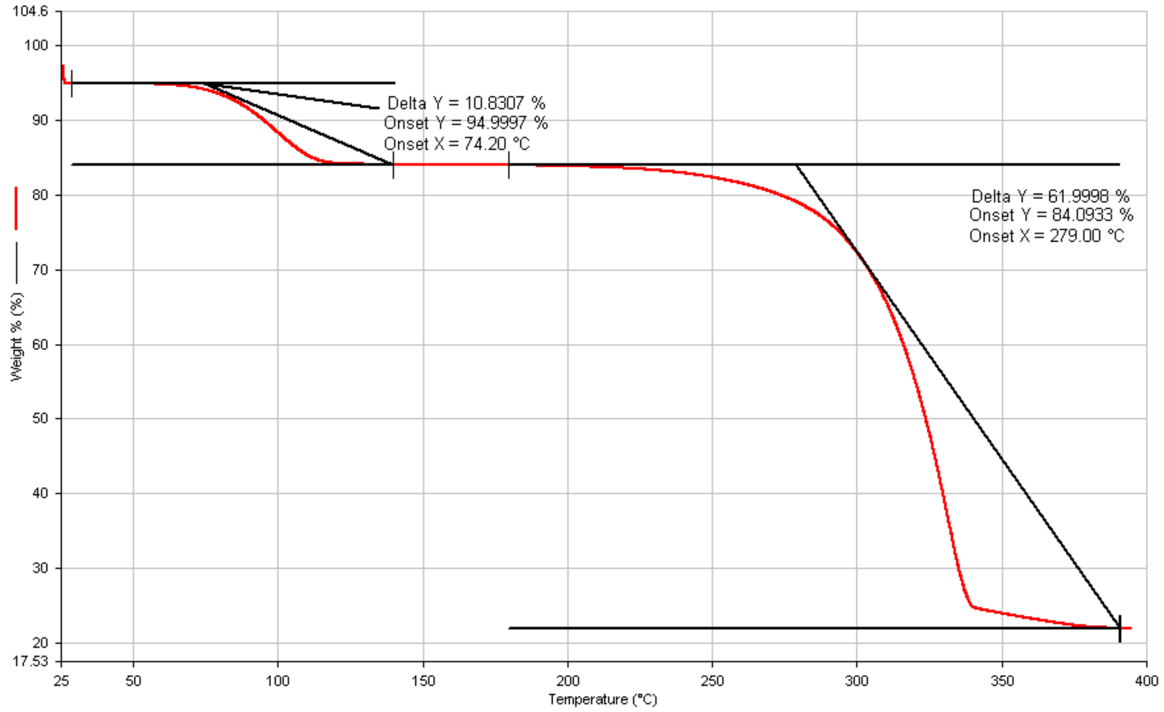

Figure S3. Thermogravimetric analysis trace and expected mass contribution table for **1-pxyl**.

[$Ag_2(O_2C(CF_2)_2CF_3)_2$(phenazine)(*m*-xylene)], 1-mxyl.

Due to the phase-impurity of samples of **1-mxyl** a consistent TGA trace could not be recorded.

3. Gas chromatographic and NMR Spectroscopic analysis of 1-tol-pxyl.tol.pxyl

Gas chromatography

The chromatogram, measured as indicated in the Experimental Section, was compared with reference chromatograms of the individual synthetic components (toluene, *p*-xylene, phenazine, silver perfluorobutyrate) as 0.07 M solutions in DMSO. These were used to identify the peak positions as shown in Figure S4. The peak areas were directly calculated and compared, giving the ratio of toluene:p-xylene 56:44.

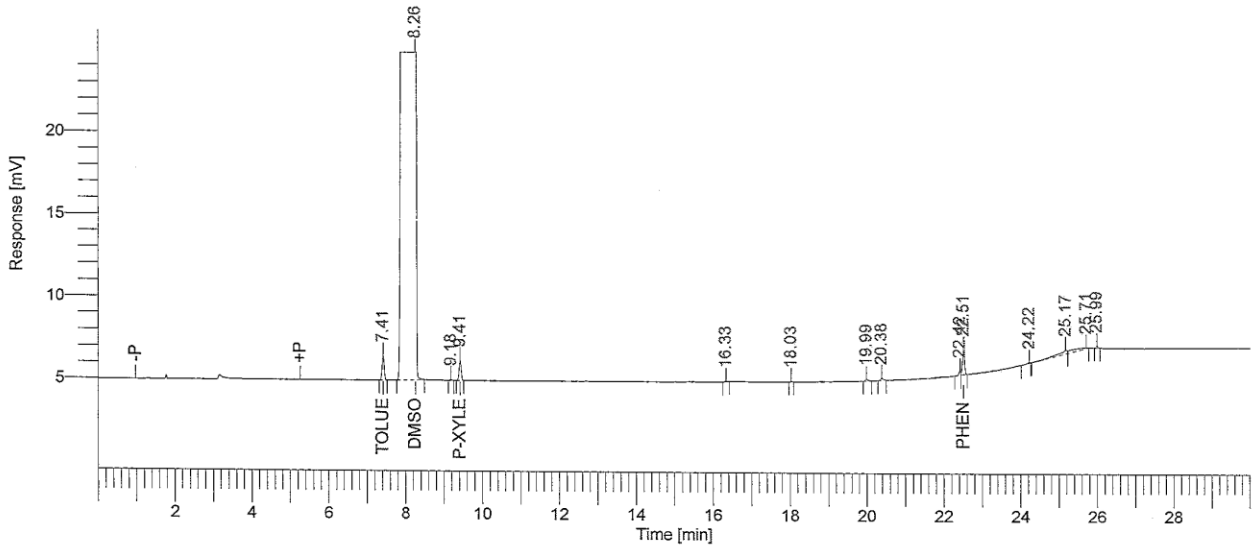

Figure S4. Gas chromatogram of **1-tol-pxyl.tol.pxyl** in DMSO.

^{1}H NMR Spectroscopy

The ^{1}H NMR spectrum, acquired as indicated in the Experimental Section, was analysed using Bruker Topspin 3.2. The integrated peak areas for the aromatic and aliphatic proton signals of toluene and *p*-xylene were compared, scaling for the different number of protons represented by each, to give an overall toluene:*p*-xylene ratio of 59:41 (Figure S5).

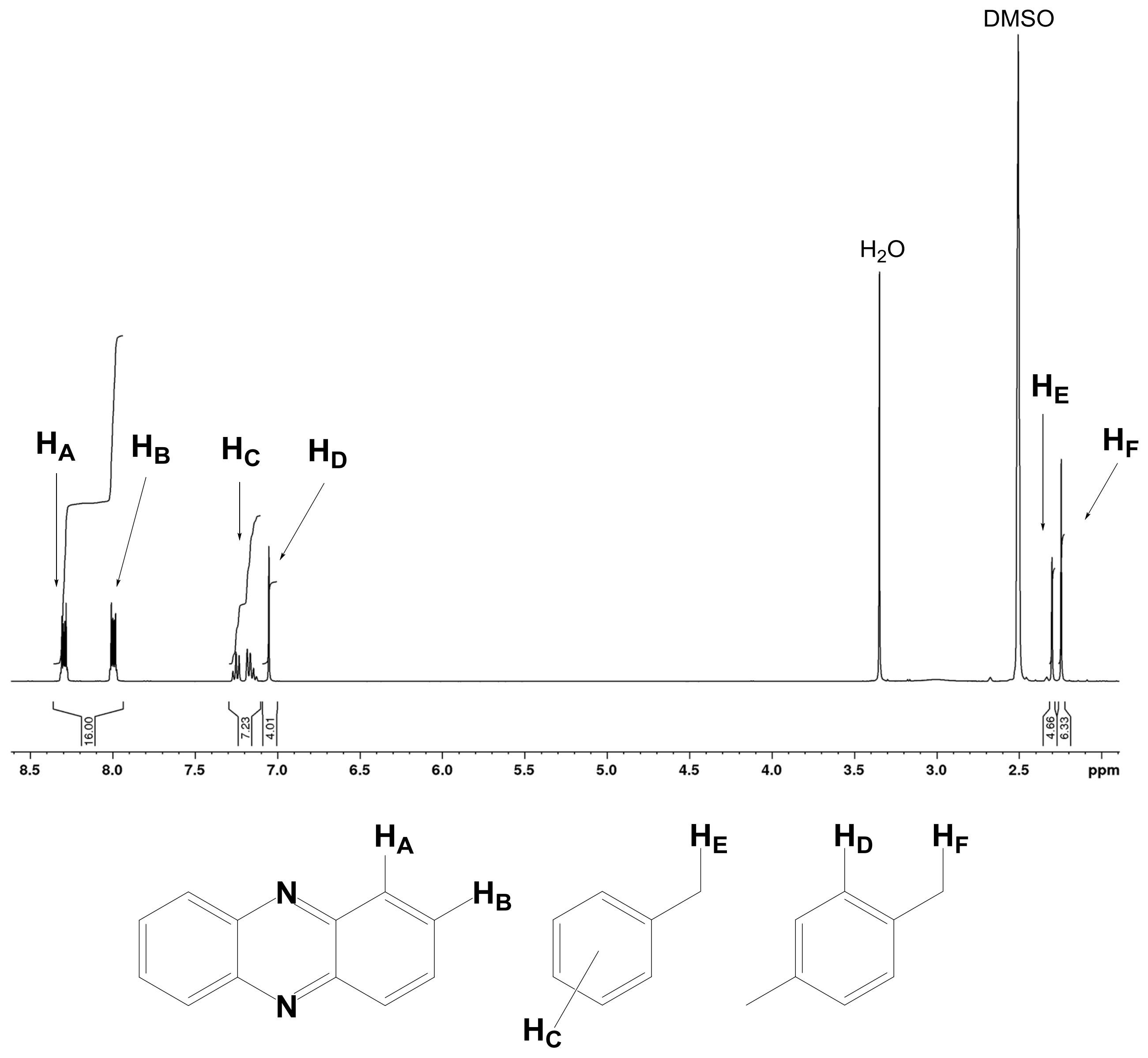

Figure S5. ^{1}H-NMR signals for aromatic and aliphatic protons in toluene and *p*-xylene and their assignments, in **1-tol-pxyl.tol.pxyl**.

4. X-ray powder diffraction studies of heating 1-tol.tol, 1-pxyl, 1-mxyl and 1-tol-pxyl.tol.pxyl

***In situ* heating study on 1-tol.tol:**

The yellow microcrystalline sample was loaded into a 0.7 mm borosilicate capillary, and the capillary end cut to allow exit of evolving vapour. The sample was heated with a co-axial stream of dry nitrogen gas using an Oxford Cryosystems Cryostream device. X-ray diffraction data were collected (λ = 0.826008 (2) Å) at beamline I11 at Diamond Light Source,[S1,S2] equipped with a wide angle (90 °) PSD detector comprising 18 Mythen-2 modules. The full study was conducted over a period of 2 hrs. At each interval (approx.. 20 mins), a pair of scans was conducted, related by a 0.25 ° detector offset to

account for gaps between detector modules. The resulting patterns were summed to give the final pattern for structural analysis. These powder patterns were analysed using the TOPAS program.[S3]

Pattern 1, 298 K. The pattern was compared with calculated X-ray powder patterns for **1-tol.tol** and **2b,** already established from single-crystal X-ray diffraction. The unit cells for these crystal structures were used as the starting point for a mixed-phase Pawley refinement,[S4] using 885 parameters (6 background, 1 zero error, 9 profile, 10 cell, 859 reflections) , resulting in final indices of fit Rwp = 9.117, Rwp' = 16.205. The starting model used for the mixed-phase Rietveld refinement,[S6] conducted using TOPAS, included the single-crystal structures of **1-tol.tol** and **2b**. Refinement employed 35 parameters (6 background, 1 zero error, 9 profile, 4 anisotropic line broadening, 12 cell, 1 scale, 2 global scale factors for thermal parameters of each structure). Rietveld refinement converged to Rwp = 13.436, Rwp' = 32.624 (Figure S6). The relative phase amounts were found to be 52.2 (7) % of **1-tol.tol** and 47.8 (7) % of **2b**.

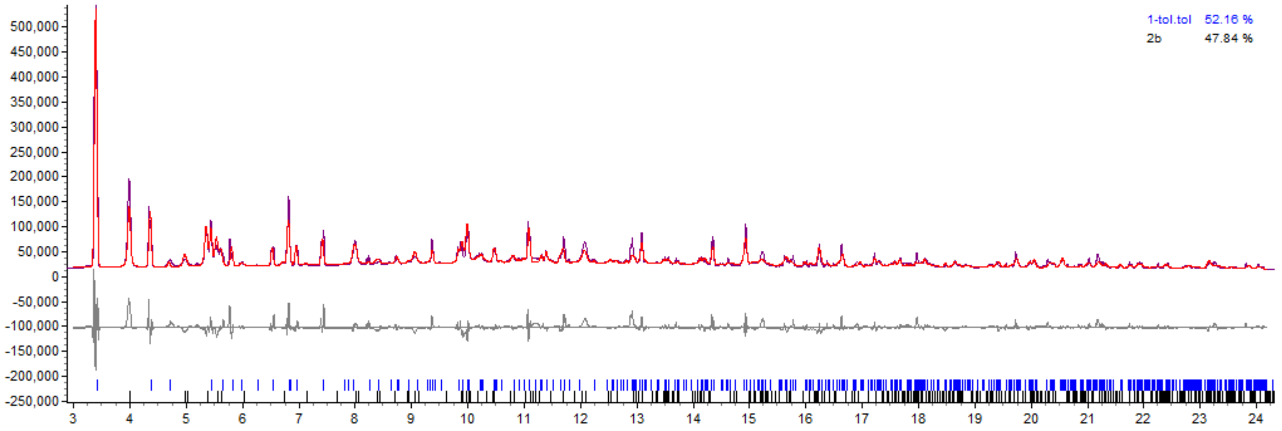

Figure S6. Observed (purple) and calculated (red) profiles and difference plot [I_{obs}-I_{calc}] (grey) of the Rietveld refinement for the sample at 298 K. (2θ range 3.0 – 24.2 °, d_{min} = 2.02 Å).

Pattern 2, sample at 313 K. As for pattern 1, the unit cells for **1-tol.tol** and **2b,** were used as the starting point for a mixed-phase Pawley refinement,[S4] using 884 parameters (6 background, 1 zero error, 9 profile, 10 cell, 858 reflections), resulting in final indices of fit Rwp = 10.955, Rwp' = 21.035. The starting model used for the mixed-phase Rietveld refinement,[S6] conducted using TOPAS, included the single-crystal structures of **1-tol.tol** and **2b**. Refinement employed 35 parameters (6 background, 1 zero error, 9 profile, 4 anisotropic line broadening, 12 cell, 1 scale, 2 global scale factors for thermal parameters of each structure). Rietveld refinement converged to Rwp = 13.862, Rwp' = 33.213 (Figure S7). The relative phase amounts were found to be 41.0 (5) % of **1-tol.tol** and 59.1 (5) % of **2b**.

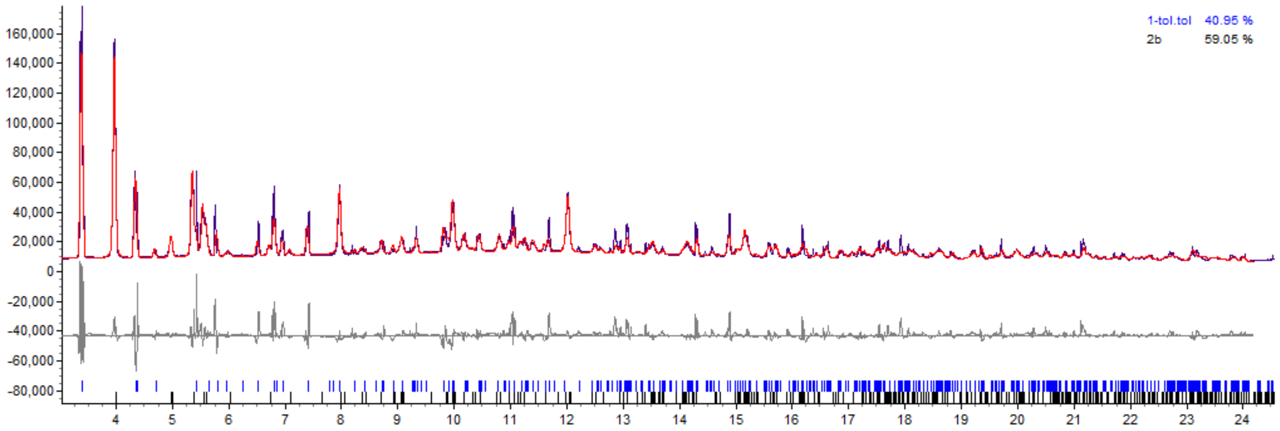

Figure S7. Observed (purple) and calculated (red) profiles and difference plot [I_{obs}-I_{calc}] (grey) of the Rietveld refinement for the sample at 313 K. (2θ range 3.0 – 24.2 °, d_{min} = 2.02 Å).

Pattern 3, sample at 330 K. As for pattern 1, the unit cells for **1-tol.tol** and **2b,** were used as the starting point for a mixed-phase Pawley refinement,[S4] using 886 parameters (6 background, 1 zero error, 9 profile, 10 cell, 860 reflections), resulting in final indices of fit Rwp = 10.258, Rwp' = 23.537. The starting model used for the mixed-phase Rietveld refinement,[S6] conducted using TOPAS, included the single-crystal structures of **1-tol.tol** and **2b**. Refinement employed 35 parameters (6 background, 1 zero error, 9 profile, 4 anisotropic line broadening, 12 cell, 1 scale, 2 global scale factors for thermal parameters of each structure). Rietveld refinement converged to Rwp = 13.713, Rwp' = 32.770 (Figure S8). The relative phase amounts were found to be 40.6 (5) % of **1-tol.tol** and 59.4 (5) % of **2b**.

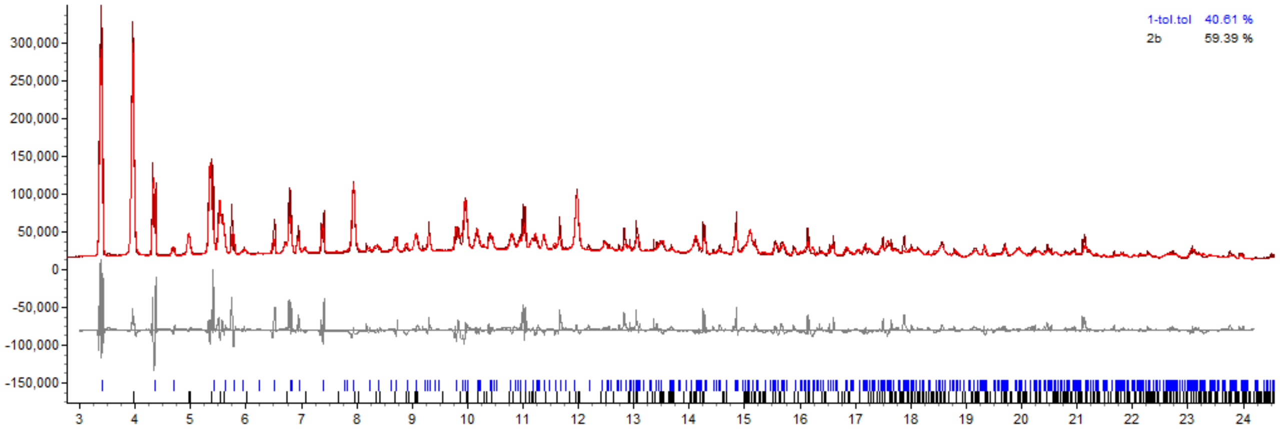

Figure S8. Observed (brown) and calculated (red) profiles and difference plot [I_{obs}-I_{calc}] (grey) of the Rietveld refinement for the sample at 330 K. (2θ range 3.0 – 24.2 °, d_{min} = 2.02 Å).

Pattern 4, sample at 350 K. As for pattern 1, the unit cells for **1-tol.tol** and **2b,** were used as the starting point for a mixed-phase Pawley refinement,[S4] using 891 parameters (6 background, 1 zero error, 9 profile, 10 cell, 865 reflections), resulting in final indices of fit Rwp = 10.258, Rwp' = 23.537. The starting model used for the mixed-phase Rietveld refinement,[S6] conducted using TOPAS, included the single-crystal structures of **1-tol.tol** and **2b**. Refinement employed 35 parameters (6 background, 1 zero error, 9 profile, 4 anisotropic line broadening, 12 cell, 1 scale, 2 global scale factors for thermal

parameters of each structure). Rietveld refinement converged to Rwp = 11.786, Rwp' = 28.372 (Figure S9). The relative phase amounts were found to be 24.5 (3) % of **1-tol.tol** and 75.5 (3) % of **2b**.

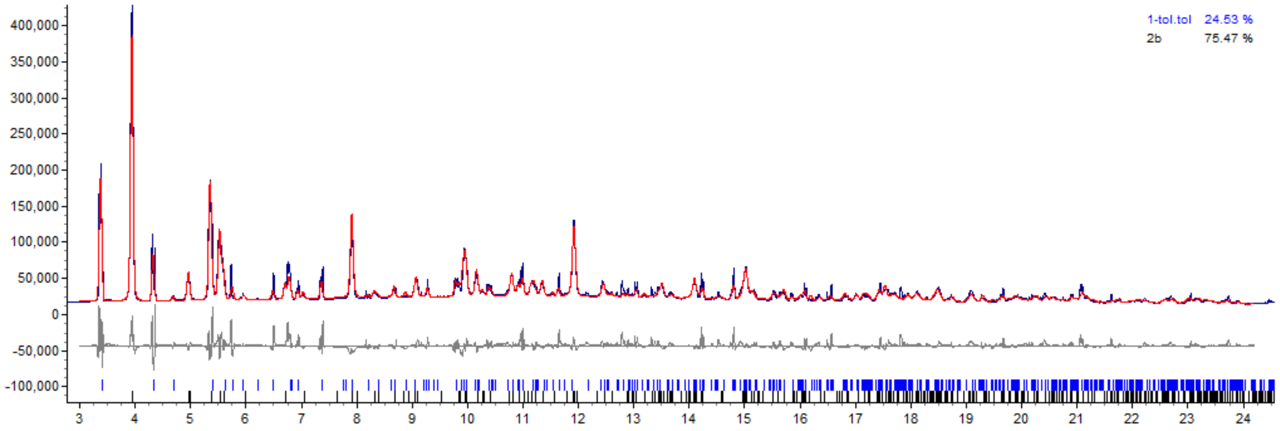

Figure S9. Observed (blue) and calculated (red) profiles and difference plot [I_{obs}-I_{calc}] (grey) of the Rietveld refinement for the sample at 350 K. (2θ range 3.0 – 24.2 °, d_{min} = 2.02 Å).

Pattern 5, sample at 373 K. The unit cell for **2b** was used as the starting point for a single-phase Pawley refinement,[S4] using 385 parameters (6 background, 1 zero error, 5 profile, 6 cell, 367 reflections), resulting in final indices of fit Rwp = 4.400, Rwp' = 10.411. The starting model used for the Rietveld refinement,[S6] conducted using TOPAS, was the single-crystal structure of **2b**. Refinement employed 24 parameters (6 background, 1 zero error, 5 profile, 4 anisotropic line broadening, 6 cell, 1 scale, 1 global scale factor for thermal parameters). Rietveld refinement converged to Rwp = 6.725, Rwp' = 16.096 (Figure S10).

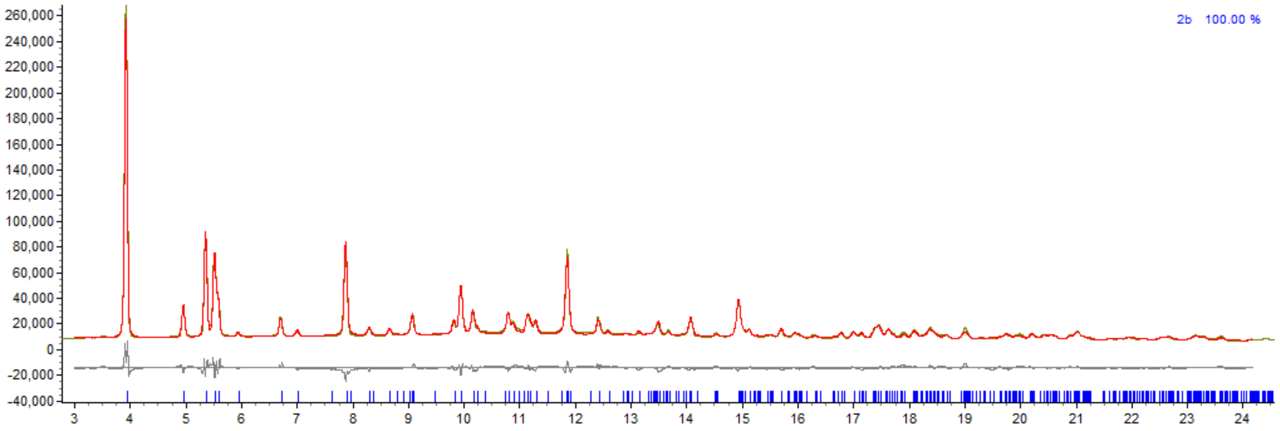

Figure S10. Observed (green) and calculated (red) profiles and difference plot [I_{obs}-I_{calc}] (grey) of the Rietveld refinement for the sample at 373 K. (2θ range 3.2 – 24.2 °, d_{min} = 2.02 Å).

Pattern 6, sample returned to 298 K. The unit cell for **2b** was used as the starting point for a single-phase Pawley refinement,[S4] using 389 parameters (6 background, 1 zero error, 5 profile, 6 cell, 371 reflections), resulting in final indices of fit Rwp = 4.563, Rwp' = 11.135. The starting model used for the Rietveld refinement,[S6] conducted using TOPAS, was the single-crystal structure of **2b**. Refinement

employed 26 parameters (6 background, 1 zero error, 5 profile, 6 anisotropic line broadening, 6 cell, 1 scale, 1 global scale factor for thermal parameters). Rietveld refinement converged to Rwp = 6.420, Rwp' = 16.437(Figure S11) .

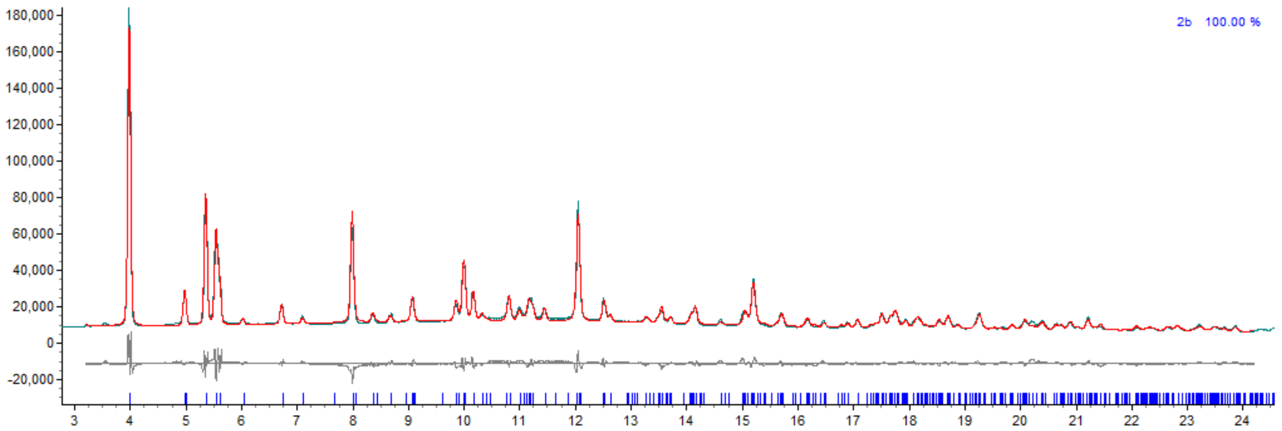

Figure S11. Observed (turquoise) and calculated (red) profiles and difference plot [I_{obs}-I_{calc}] (grey) of the Rietveld refinement for the sample returned to 298 K. (2θ range 3.2 – 24.2 °, d_{min} = 2.02 Å).

***In situ* heating study on 1-pxyl:**

The yellow microcrystalline sample was loaded into a 0.7 mm borosilicate capillary, and the capillary end cut to allow exit of evolving vapour. The sample was heated with a co-axial stream of dry nitrogen gas using an Oxford Cryosystems Cryostream device. X-ray diffraction data were collected (λ = 0.826136(2) Å) at beamline I11 at Diamond Light Source,[S1,S2] equipped with a wide angle (90 °) PSD detector comprising 18 Mythen-2 modules. At each interval, as indicated below, a pair of scans was conducted, related by a 0.25 ° detector offset to account for gaps between detector modules. The resulting patterns were summed to give the final pattern for structural analysis. These powder patterns were analysed using the TOPAS program.[S3]

Pattern 1, 298 K. The pattern was compared with the calculated X-ray powder pattern for **1-pxyl,** whose structure already established from single-crystal X-ray diffraction. This unit cell was used for a Pawley refinement,[S4] employing 1515 parameters (8 background, 1 zero error, 5 profile, 4 cell, 1497 reflections), and resulting in final indices of fit Rwp = 4.602, Rwp' = 9.192. The starting model used for the Rietveld refinement,[S6] conducted using TOPAS, was the single-crystal structure of **1-pxyl**. Refinement employed 20 parameters (8 background, 1 zero error, 5 profile, 4 cell, 1 scale, 1 global scale factor for thermal parameters). Rietveld refinement converged to Rwp = 12.026, Rwp' = 22.338 (Figure S12).

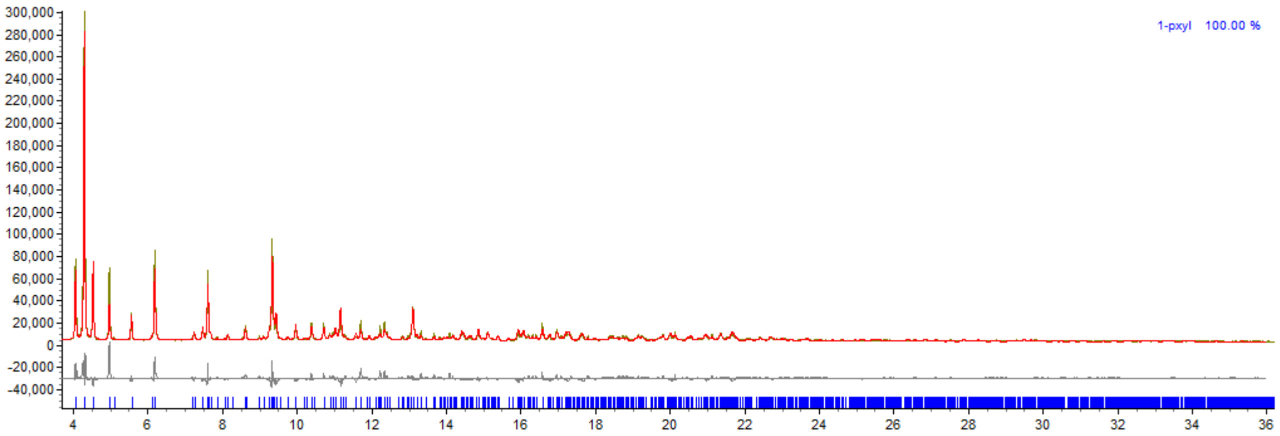

Figure S12. Observed (green) and calculated (red) profiles and difference plot [I_{obs}-I_{calc}] (grey) of the Rietveld refinement. (2θ range 3.5 - 36 °, d_{min} = 1.41 Å).

Pattern 2, 10 min at 373 K. The pattern was compared with calculated X-ray powder patterns for **1-pxyl, 2a** and **2b**, crystal structures of which were already established from single-crystal X-ray diffraction. The unit cells for these crystal structures were used as the starting point for a mixed-phase Pawley refinement,[S4] using 3895 parameters (6 background, 1 zero error, 13 profile, 14 cell, 3861 reflections), resulting in final indices of fit Rwp = 7.763, Rwp' = 15.183. The starting model used for the Rietveld refinement,[S6] conducted using TOPAS, included the single-crystal structures of **1-pxyl**, **2a** and **2b**. Refinement employed 38 parameters (6 background, 1 zero error, 13 profile, 14 cell, 1 scale, 3 global scale factors for thermal parameters of each structure). Rietveld refinement converged to Rwp = 12.549, Rwp' = 26.006 (Figure S13). The refinement found the material to contain **1-pxyl** 91.3 (5) %, **2a** 6.6 (3) % and **2b** 1.8 (5) %.

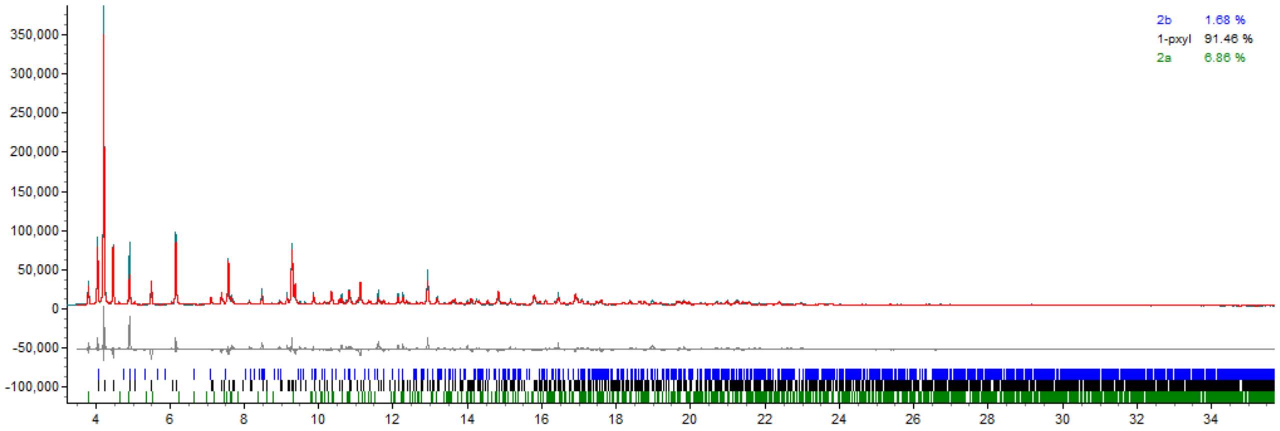

Figure S13. Observed (turquoise) and calculated (red) profiles and difference plot [I_{obs}-I_{calc}] (grey) of the Rietveld refinement after 10 min at 373 K. (2θ range 3.5 - 36 °, d_{min} = 1.41 Å).

Pattern 3, 20 min at 373 K. As for pattern 2, the unit cells for **1-pxyl, 2a** and **2b** were used as the starting point for a mixed-phase Pawley refinement,[S4] using 3913 parameters (8 background, 1 zero error, 13 profile, 14 cell, 3877 reflections), resulting in final indices of fit Rwp = 4.217, Rwp' = 9.354.

The starting models used for the Rietveld refinement,[S6] conducted using TOPAS, included the single-crystal structures of **1-pxyl**, **2a** and **2b**. Refinement employed 40 parameters (8 background, 1 zero error, 13 profile, 14 cell, 1 scale, 3 global scale factors for thermal parameters of each structure). Rietveld refinement converged to Rwp = 11.587, Rwp' = 26.255 (Figure S14). The refinement found the material to contain **1-pxyl** 20.9 (1) %, **2a** 72.7 (2) % and **2b** 6.4 (1) %.

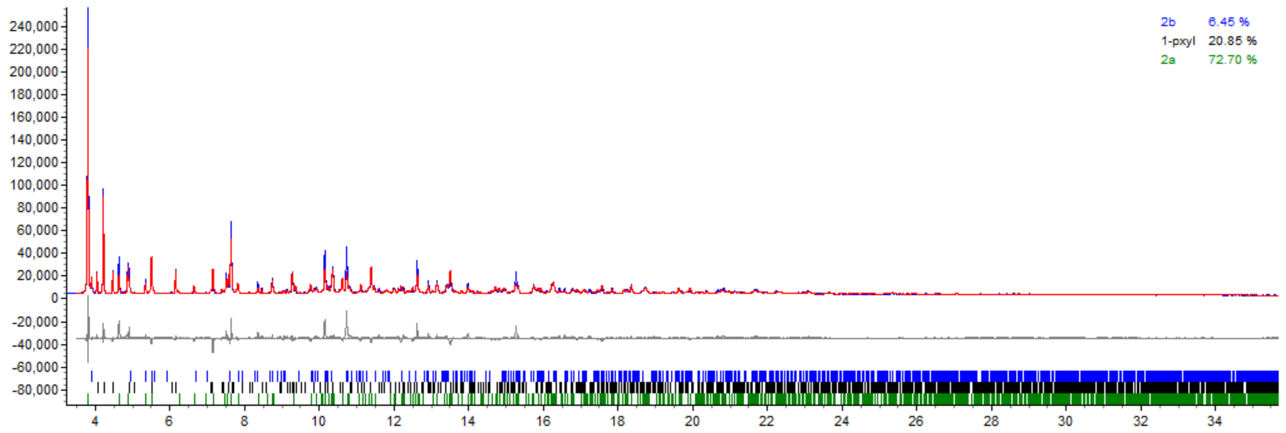

Figure S14. Observed (blue) and calculated (red) profiles and difference plot [I_{obs}-I_{calc}] (grey) of the Rietveld refinement after 20 min at 373 K. (2θ range 3.5 - 36 °, d_{min} = 1.41 Å).

Pattern 4, 30 min at 373 K. As for pattern 2, the unit cells for **1-pxyl, 2a** and **2b** were used as the starting point for a mixed-phase Pawley refinement,[S4] using 3913 parameters (8 background, 1 zero error, 13 profile, 14 cell, 3877 reflections), resulting in final indices of fit Rwp = 5.304, Rwp' = 12.584. The starting models used for the Rietveld refinement,[S6] conducted using TOPAS, included the single-crystal structures of **1-pxyl**, **2a** and **2b**. Refinement employed 40 parameters (8 background, 1 zero error, 13 profile, 14 cell, 1 scale, 3 global scale factors for thermal parameters of each structure). Rietveld refinement converged to Rwp = 11.590, Rwp' = 26.322 (Figure S15) . The refinement found the material to contain **1-pxyl** 1.2 (1) %, **2a** 81.4 (3) % and **2b** 17.5 (2) %.

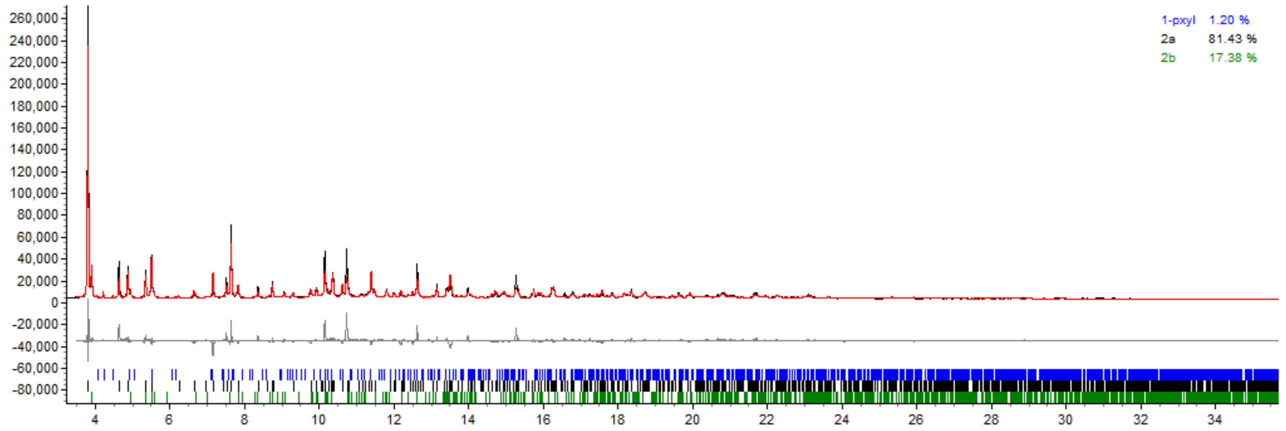

Figure S15. Observed (black) and calculated (red) profiles and difference plot [I_{obs}-I_{calc}] (grey) of the Rietveld refinement after 30 min at 373 K. (2θ range 3.5 - 36 °, d_{min} = 1.41 Å).

Pattern 5, 40 min at 373 K. The unit cells for **2a** and **2b** were used as the starting point for a mixed-phase Pawley refinement,[S4] using 2365 parameters (8 background, 1 zero error, 9 profile, 10 cell, 2337 reflections), resulting in final indices of fit Rwp = 4.264, Rwp' = 9.687. The starting model used for the mixed-phase Rietveld refinement,[S6] conducted using TOPAS, included the single-crystal structures of **2a** and **2b**. Refinement employed 31 parameters (8 background, 1 zero error, 9 profile, 10 cell, 1 scale, 2 global scale factors for thermal parameters of each structure). Rietveld refinement converged to Rwp = 11.660, Rwp' = 26.516 (Figure S16). The relative phase amounts were found to be 81.9 (2) % of **2a** and 18.1 (2) % of **2b**.

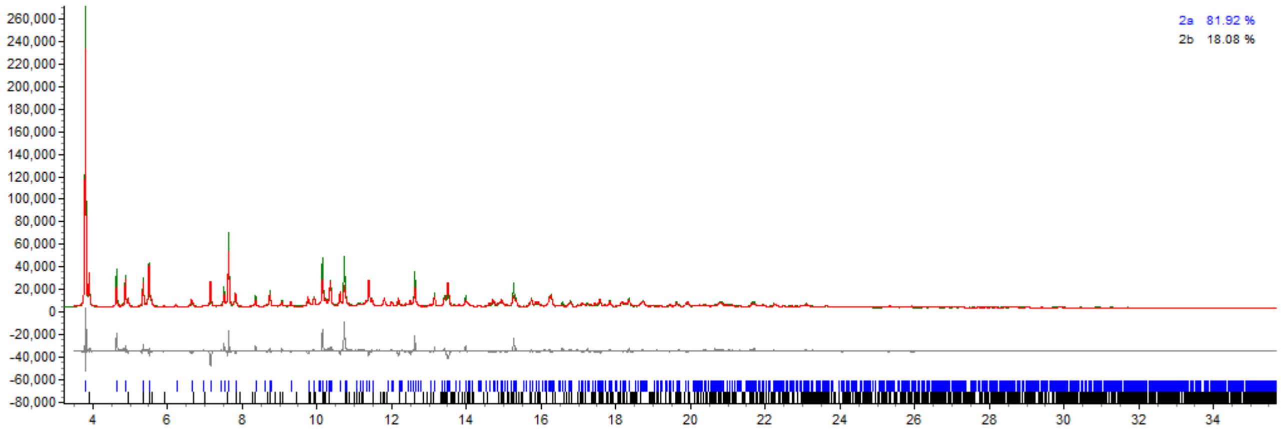

Figure S16. Observed (green) and calculated (red) profiles and difference plot [I_{obs}-I_{calc}] (grey) of the Rietveld refinement after 40 min at 373 K. (2θ range 3.5 - 36 °, d_{min} = 1.41 Å).

Pattern 6, returned to 298 K. The unit cells for **2a** and **2b** were used as the starting point for a mixed-phase Pawley refinement,[S4] using 1134 parameters (10 background, 1 zero error, 9 profile, 10 cell, 1104 reflections), resulting in final indices of fit Rwp = 4.264, Rwp' = 9.687. The starting model used for the mixed-phase Rietveld refinement,[S6] conducted using TOPAS, included the single-crystal structures of **2a** and **2b**. Refinement employed 33 parameters (10 background, 1 zero error, 9 profile, 10 cell, 1 scale, 2 global scale factors for thermal parameters of each structure) Rietveld refinement converged to Rwp = 10.929, Rwp' = 23.824 (Figure S17). The relative phase amounts were found to be 82.8 (3) % of **2a** and 17.2 (2) % of **2b**.

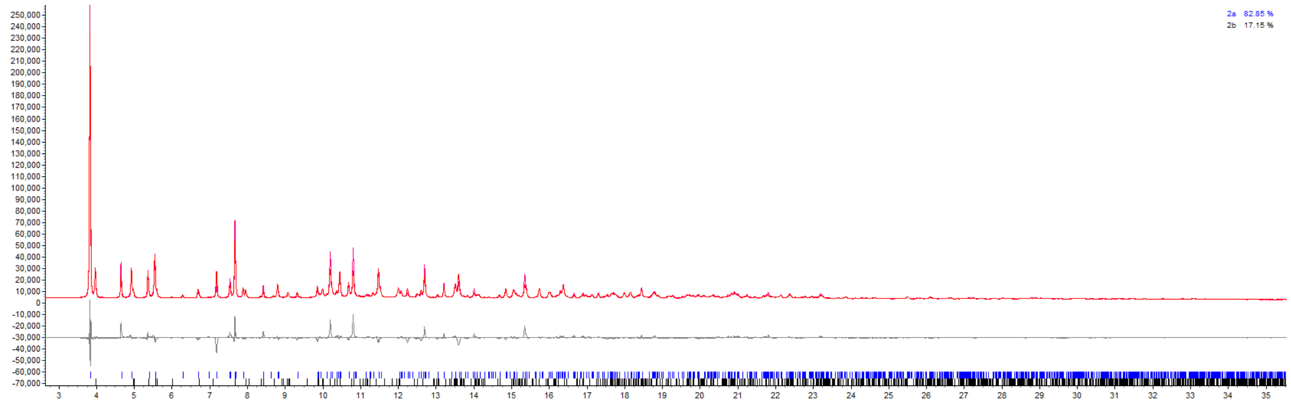

Figure S17. Observed (pink) and calculated (red) profiles and difference plot [I_{obs}-I_{calc}] (grey) of the Rietveld refinement after returning the sample to 298 K. (2θ range 3.5 - 36 °, d_{min} = 1.41 Å).

***In situ* heating study on 1-mxyl:**

The yellow microcrystalline sample was loaded into a 0.7 mm borosilicate capillary, and the capillary end cut to allow exit of evolving vapour. The sample was heated with a co-axial stream of dry nitrogen gas using an Oxford Cryosystems Cryostream device. X-ray diffraction data were collected (λ = 0.826136(2) Å) at beamline I11 at Diamond Light Source,[S1,S2] equipped with a wide angle (90 °) PSD detector comprising 18 Mythen-2 modules. At each interval, as indicated below, a pair of scans was conducted, related by a 0.25 ° detector offset to account for gaps between detector modules. The resulting patterns were summed to give the final patterns for structural analysis (Figure S18). These powder patterns were analysed using the TOPAS program.[S3] Multi-phase Pawley refinement for each of the patterns during the *in situ* was conducted, but full Rietveld refinement was not possible. Thus, the relative composition of each component could not be determined quantitatively.

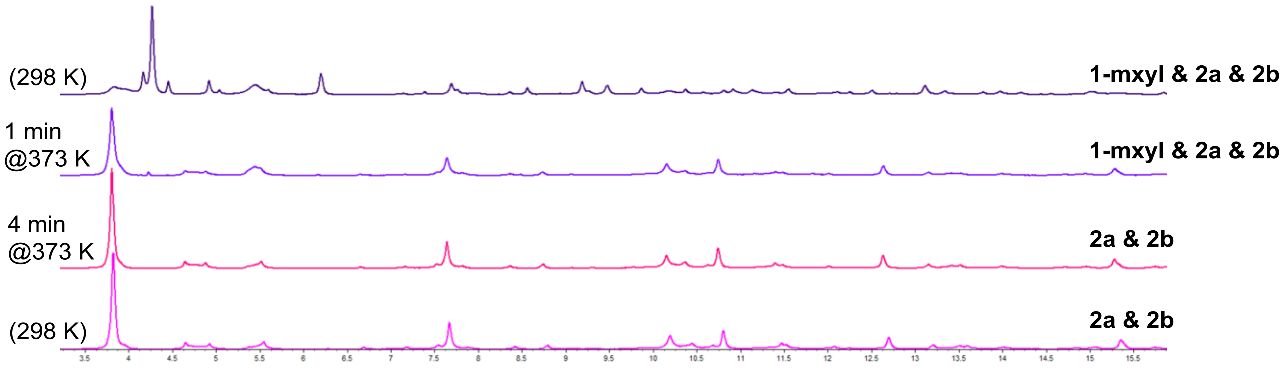

Figure S18. *In situ* X-ray powder diffraction heating study showing the conversion of **1-mxyl** to a mixture of **2a** and **2b**.

Pattern 1, 298 K. The pattern was compared with calculated X-ray powder patterns for **1-mxyl, 2a** and **2b**, crystal structures of which were already established from single-crystal X-ray diffraction. The unit cells for these crystal structures were used as the starting point for a mixed-phase Pawley

refinement,[S4] using 2724 parameters (11 background, 1 zero error, 13 profile, 14 cell, 2685 reflections). Pawley refinement converged to Rwp = 2.472, Rwp' = 4.130 (Figure S19).

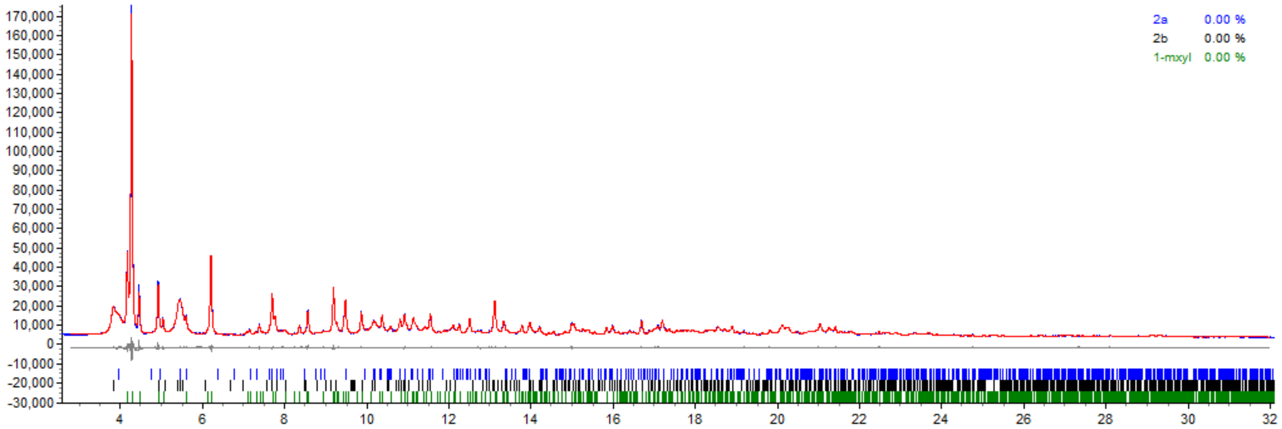

Figure S19. Observed (blue) and calculated (red) profiles and difference plot [I_{obs}-I_{calc}] (grey) of the Pawley refinement of the sample at 298 K. (2θ range 3.5 – 32.5 °, d_{min} = 1.48 Å).

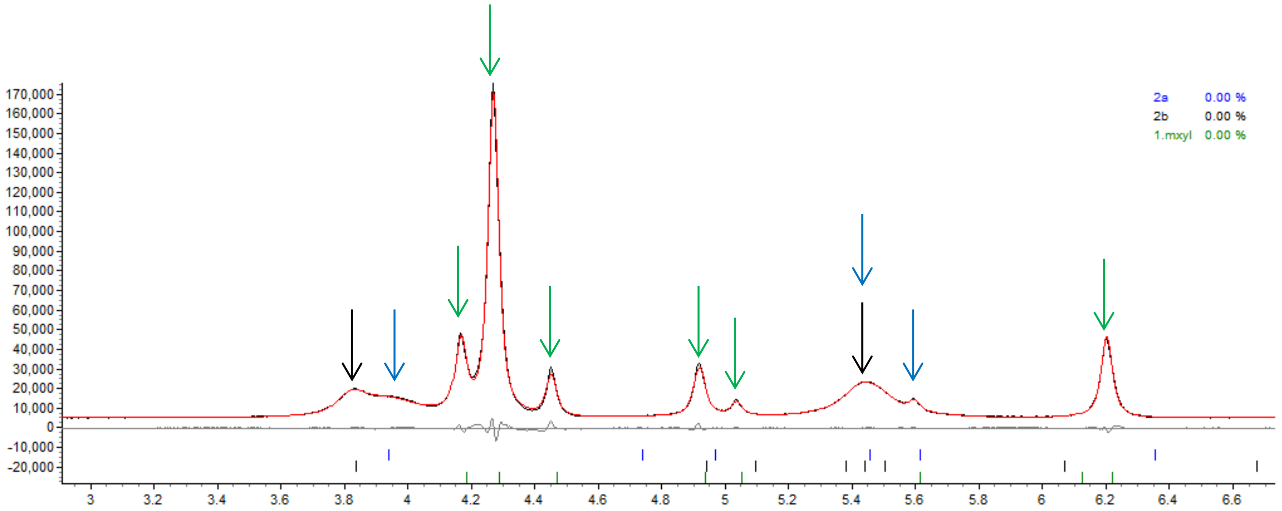

Figure S20. Low angle data from Pawley fit shown in Figure S19. Colour-coded tickmarks and arrows indicate peaks characteristic of the three phases present: **1-mxyl** (green), **2a** (blue) and **2b** (black).

Pattern 2, 1 min @ 373 K. As for pattern 1, the unit cells for **1-mxyl, 2a** and **2b** were used as the starting point for a mixed-phase Pawley refinement,[S4] using 3933 parameters (7 background, 1 zero error, 13 profile, 14 cell, 3898 reflections). Pawley refinement converged to Rwp = 2.455, Rwp' = 6.196 (Figure S21).

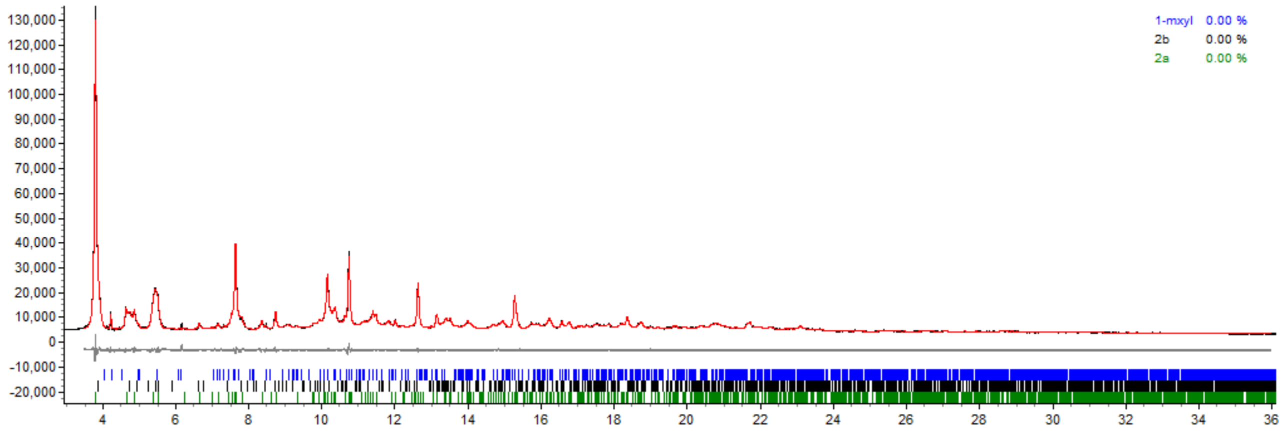

Figure S21. Observed (black) and calculated (red) profiles and difference plot $[I_{obs}-I_{calc}]$ (grey) of the Pawley refinement of the sample after 1 min at 373 K. (2θ range 3.5 - 36 °, d_{min} = 1.41 Å).

Pattern 3, 4 min @ 373 K. The unit cells for **2a** and **2b** were used as the starting point for a mixed-phase Pawley refinement,[S4] using 2402 parameters (7 background, 1 zero error, 9 profile, 10 cell, 2375 reflections). Pawley refinement converged to Rwp = 4.193, Rwp' = 9.174 (Figure S22).

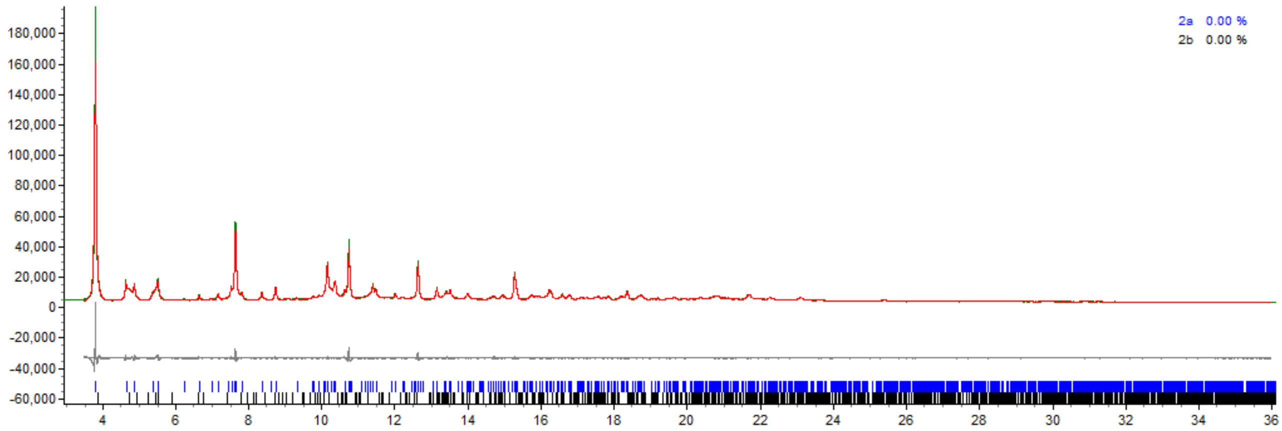

Figure S22. Observed (green) and calculated (red) profiles and difference plot $[I_{obs}-I_{calc}]$ (grey) of the Pawley refinement of the sample after 4 min at 373 K. (2θ range 3.5 - 36 °, d_{min} = 1.41 Å).

Pattern 4, returned to 298 K. The unit cells for **2a** and **2b** were used as the starting point for a mixed-phase Pawley refinement,[S4] using 1137 parameters (9 background, 1 zero error, 9 profile, 10 cell, 1108 reflections). Pawley refinement converged to Rwp = 5.831, Rwp' = 14.323 (Figure S23).

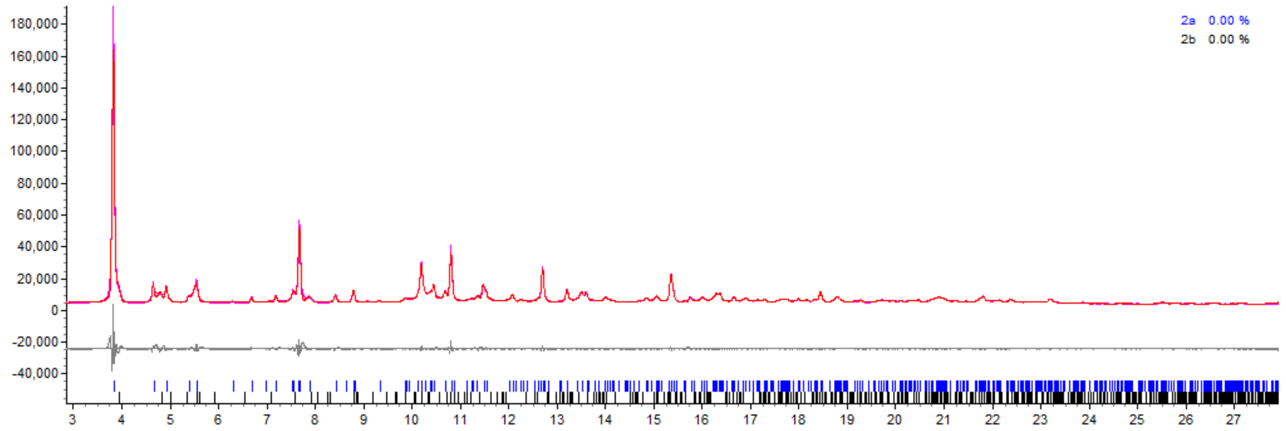

Figure S23. Observed (pink) and calculated (red) profiles and difference plot [I_{obs}-I_{calc}] (grey) of the Pawley refinement of the sample after returning to 298 K. (2θ range 3.5 – 28.5 °, d_{min} = 1.68 Å).

Although Rietveld refinement on the resultant diffraction pattern for this material could not be conducted, visual comparison with the pattern for material resultant from heating **1-pxl** indicates a similar composition (i.e. **2a** and **2b**), as established qualitatively by Pawley refinement (see Figure 24).

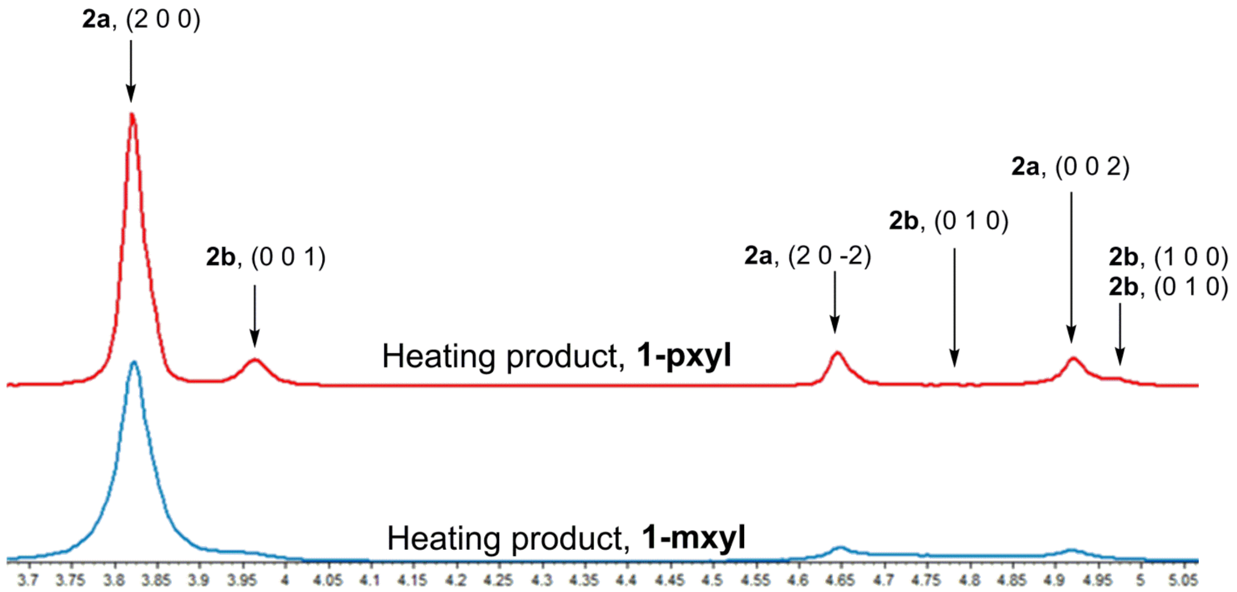

Figure S24. Comparison of the low-angle data, showing the key reflections for **2a** and **2b**, in the materials resultant from the heating studies on **1-pxyl** (pattern that shown in Figure S17, shown here in red) and **1-mxyl** (pattern that shown in Figure S23, shown here in blue). Reflections are labelled appropriately.

Phase-purity of / guest loss by 1-tol-pxyl.tol.pxyl. The yellow microcrystalline **1-tol-pxyl.tol.pxyl** was dried in air for 30 minutes and loaded into a 0.7 mm borosilicate capillary. X-ray diffraction data were collected (λ = 0.826136(2) Å) at beamline I11 at Diamond Light Source,[S1,S2] equipped with a wide angle (90 °) PSD detector comprising 18 Mythen-2 modules. A pair of scans was conducted at room

temperature, related by a 0.25 ° detector offset to account for gaps between detector modules. The resulting patterns were summed to give the final pattern for structural analysis. The powder pattern was indexed using the TOPAS program.[S3] Although some peaks indexed matched those corresponding to the known phases **1-tol-pxyl.tol.pxyl** and **2b**, other peaks which could not be matched to any of the other known phases described in this paper were present. A Pawley refinement (Figure S25) was conducted by starting with unit cell parameters matching those of **1-tol-pxyl.tol.pxyl** and **2b**, using 264 parameters (12 background, 1 zero error, 9 profile, 12 cell, 230 reflections). (Rwp = 14.903, Rwp' = 32.946).[S4] **1-tol-pxyl.tol.pxyl:** [a = 10.656 (2) Å, b = 11.259 (2) Å, c = 14.573 (2) Å, α = 72.62 (2) °, β = 84.13 (1) °, γ = 83.07 (2) °, V = 1652.2 (5) $Å^3$]. **2b:** [a = 10.82 (1) Å, b = 10.98 (1) Å, c = 12.616 (7) Å, α = 70.49 (7) °, β = 75.57 (6) °, γ = 62.60 (6) °, V = 1246 (2) $Å^3$].

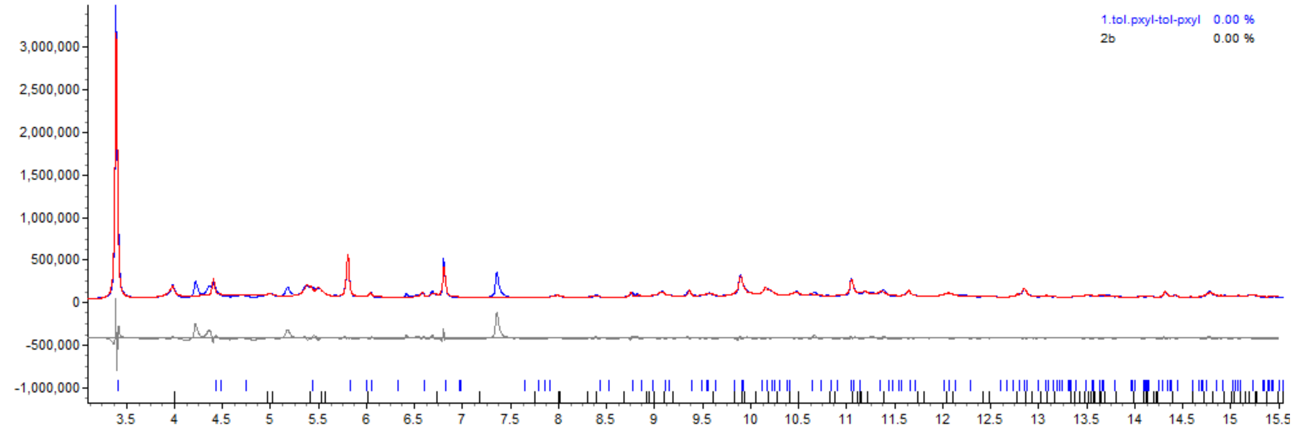

Figure S25. Observed (blue) and calculated (red) profiles and difference plot [I_{obs}-I_{calc}] (grey) of the Pawley refinement. (2θ range 3.0 – 15.5 °, d_{min} = 3.10 Å).

5. Phase identification by X-ray powder diffraction from *ex situ* vapour exposure experiments

Phase identification: 1-tol.tol exposed to *o*-xylene vapour. The yellow microcrystalline product was loaded into a 0.5 mm borosilicate capillary. X-ray diffraction data were collected (λ = 0.3997939(15) Å) at station ID31 at the European Synchrotron Radiation Facility,[S5] using a 9-channel multi-crystal analyser crystal (MAC) detector. All data were collected at room temperature. The powder pattern was indexed using the TOPAS program.[S3] The pattern was compared with calculated X-ray powder patterns for polymorphs **2a** and **2b** already established from single-crystal X-ray diffraction. The unit cell parameters of these compounds were used as the starting point for a mixed-phase Pawley refinement[S4] (Figure S26), employing 689 parameters (7 background, 1 zero error, 9 profile, 10 cell, 662 reflections). Pawley refinement converged to Rwp = 12.902, Rwp' = 27.943. **2a:** [a = 27.7545 (4) Å, b = 9.3749 (1) Å, c = 21.5607 (2) Å, β = 117.389 (1) °, V = 4981.2 (1) $Å^3$]. **2b:** [a =

10.7983 (6) Å, b = 10.9939 (7) Å, c = 12.5520 (5) Å, α = 71.506 (6) °, β = 76.039 (6) °, γ = 62.311 (5) °, V = 1243.5 (1) Å^3].

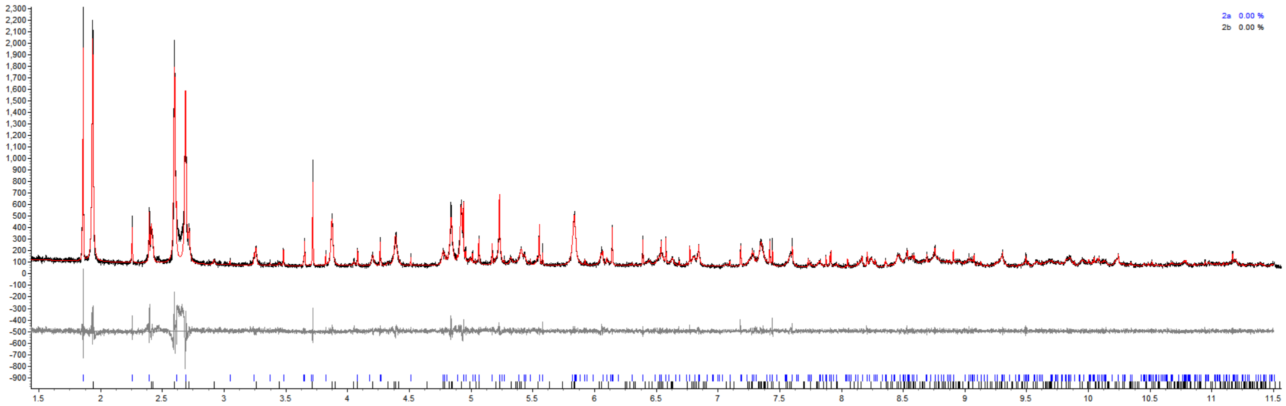

Figure S26. Observed (black) and calculated (red) profiles and difference plot [I_{obs}-I_{calc}] (grey) of the Pawley refinement. (2θ range 1.4 – 11.5 °, d_{min} = 2.00 Å).

Phase identification, 1-tol.tol exposed to *m*-xylene vapour. The yellow microcrystalline product was loaded into a 0.5 mm borosilicate capillary. X-ray diffraction data were collected (λ = 0.3997939(15) Å) at station ID31 at the European Synchrotron Radiation Facility,[S5] using a 9-channel multi-crystal analyser crystal (MAC) detector. All data were collected at room temperature. The powder pattern was indexed using the TOPAS program.[S3] The pattern was compared with calculated X-ray powder pattern for polymorph **2a,** already established from single-crystal X-ray diffraction. The unit cell parameters for **2a** were used as the starting point for a Pawley refinement[S4] (Figure S27), employing 431 parameters (7 background, 1 zero error, 5 profile, 4 cell, 414 reflections). Pawley refinement converged to Rwp = 11.574, Rwp' = 22.047. [a = 27.7573 (1) Å, b = 9.37554 (4) Å, c = 21.56215 (8) Å, β = 117.3816 (4) °, V = 4981.65 (4) Å^3].

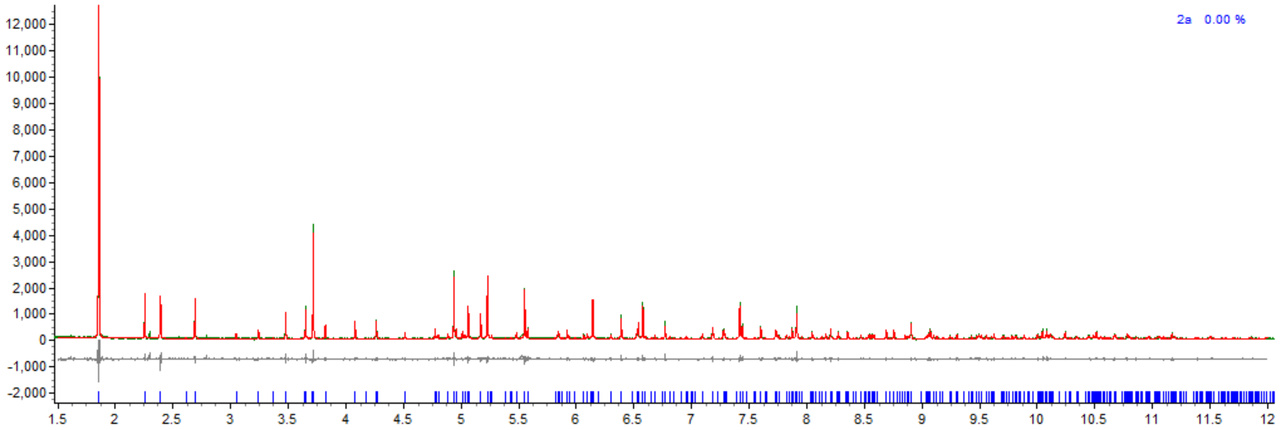

Figure S27. Observed (green) and calculated (red) profiles and difference plot [I_{obs}-I_{calc}] (grey) of the Pawley refinement. (2θ range 1.5 – 12.0 °, d_{min} = 1.92 Å).

Phase identification: 1-tol.tol exposed to *p*-xylene vapour. The yellow microcrystalline product was loaded into a 0.5 mm borosilicate capillary. X-ray diffraction data were collected (λ = 0.3997939(15) Å) at station ID31 at the European Synchrotron Radiation Facility,[S5] using a 9-channel multi-crystal analyser crystal (MAC) detector. All data were collected at room temperature. The powder pattern was indexed using the TOPAS program.[S3] The pattern was compared with calculated X-ray powder patterns for polymorphs **2a** and **2b** and the *p*-xylene-containing structure **1-pxyl**, already established from single-crystal X-ray diffraction. The unit cell parameters of these three materials were used as a starting point for a mixed-phase Pawley refinement[S4] (Figure S28), employing 156 parameters (6 background, 1 zero error, 13 profile, 14 cell, 122 reflections). Pawley refinement converged to Rwp = 24.770, Rwp' = 54.817. **1-pxyl:** [a = 11.15 (1) Å, b = 23.216 (2) Å, c = 13.35 (2) Å, β = 110.8 (2) °, V = 3231 (7) Å^3]. **2a:** [a = 27.680 (2) Å, b = 9.3757 (6) Å, c = 21.572 (1) Å, β = 117.102 (6) °, V = 4983.8 (6) Å^3]. **2b:** [a = 10.813 (1) Å, b = 10.971 (1) Å, c = 12.491 (2) Å, α = 72.17 (2) °, β = 76.58 (2) °, γ = 62.15 (1) °, V = 1240.3 (3) Å^3]. It should be noted that the component modelled here as **1-pxyl**, could also be an isostructural compound **1-tol** or **1-tol-pxyl**, as yet not independently identified. A fourth, unidentified phase is also present, as noted in the Scheme 3 and the corresponding discussion.

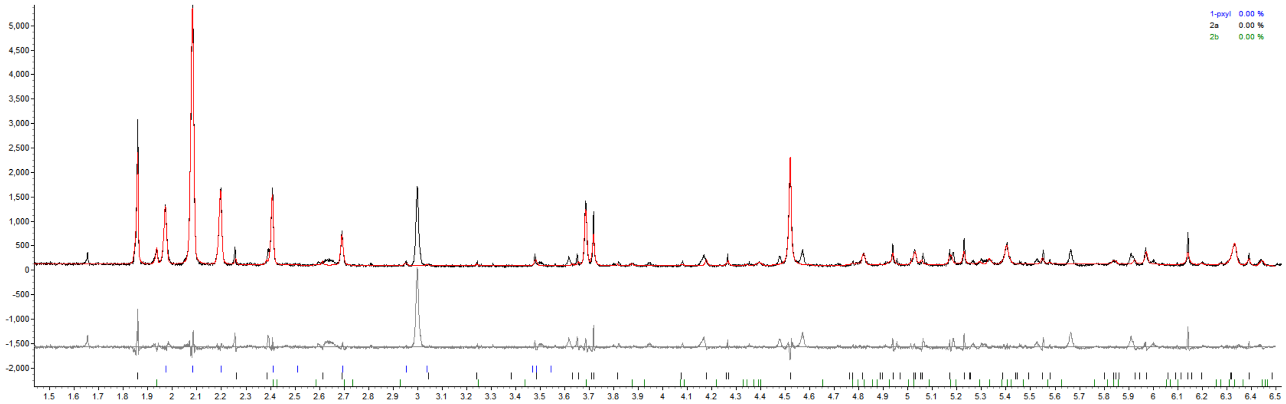

Figure S28. Observed (black) and calculated (red) profiles and difference plot [I_{obs}-I_{calc}] (grey) of the Pawley refinement. (2θ range 1.4 – 6.5 °, d_{min} = 3.53 Å).

Phase identification: 2b exposed to methanol vapour. The yellow microcrystalline product was loaded into a 0.5 mm borosilicate capillary. X-ray diffraction data were collected (λ = 1.5406 Å) at University of Sheffield, Department of Materials Science on a STOE STADI P $Cu_{K\alpha}$. Data were collected on a PSD detector with a single scan (5 < 2θ < 40 °) at a scan rate of 0.067 ° min^{-1}, using a rotating capillary. The powder pattern was indexed using the TOPAS program.[3] The pattern was compared with calculated X-ray powder patterns for polymorphs **2a** and **2b**, already established from single-crystal X-ray diffraction. The unit cell parameters for these compounds were used as a starting point for a mixed-phase Pawley refinement,[S4] employing 795 parameters (6 background, 1 zero error, 9 profile, 10 cell, 769 reflections), resulting in final indices of fit Rwp = 3.095, Rwp' = 9.023. The starting

models used for the mixed-phase Rietveld refinement,[S6] conducted using TOPAS, were the single-crystal structures of **2a** and **2b**. The model for the structure was refined using one global isotropic thermal parameter. The sample was found to contain 34.3 (3) % **2a** and 65.7 (3) % **2b** by Rietveld fitting (Figure S29), using 28 parameters (6 background, 1 zero error, 9 profile, 10 cell, 1 scale, 1 global scale factor for thermal parameters). Rietveld refinement converged to Rwp = 6.111, Rwp' = 20.839. **2a:** [*a* = 27.40 (8) Å, *b* = 8.78 (3) Å, *c* = 20.45 (7) Å, β = 117.6 (3) °, *V* = 4361 (27) Å^3]. **2b:** [*a* = 10.7684 (8) Å, *b* = 10.9844 (8) Å, *c* = 12.5309 (8) Å, α = 71.593 (5) °, β = 76.126 (6) °, γ = 62.318 (5) °, *V* = 1237.7 (2) Å^3].

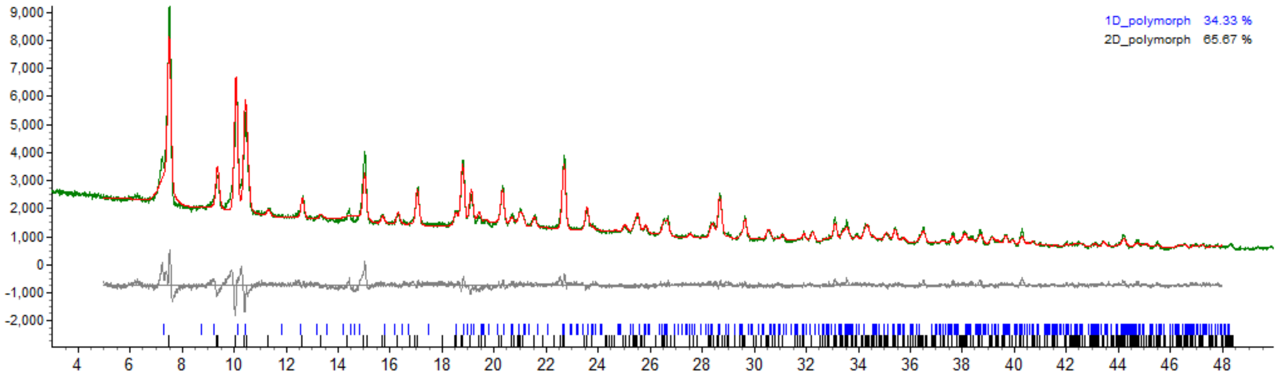

Figure S29. Observed (green) and calculated (red) profiles and difference plot [I_{obs}-I_{calc}] (grey) of the Rietveld refinement. (2θ range 5.0 – 48.0 °, d_{min} = 2.07 Å).

Phase identification: 2b exposed to ethanol vapour. The yellow microcrystalline product was loaded into a 0.5 mm borosilicate capillary. X-ray diffraction data were collected (λ = 1.5406 Å) at University of Sheffield, Department of Materials Science on a STOE STADI P $Cu_{K\alpha}$. Data were collected on a PSD detector with a single scan (5 < 2θ < 40 °) at a scan rate of 0.067 ° min^{-1}, using a rotating capillary. The powder pattern was indexed using the TOPAS program.[S3] The pattern was compared with calculated X-ray powder patterns for polymorphs **2a** and **2b**, already established from single-crystal X-ray diffraction. The unit cell parameters for these compounds were used as a starting point for a mixed-phase Pawley refinement,[S4] employing 723 parameters (6 background, 1 zero error, 9 profile, 10 cell, 697 reflections), and resulting in final indices of fit Rwp = 3.431, Rwp' = 9.880. The starting models used for the mixed-phase Rietveld refinement,[S6] conducted using TOPAS, were the single-crystal structures of **2a** and **2b**. The model for the structure was refined using one global isotropic thermal parameter. The sample was found to contain 38.0 (2) % **2a** and 62.0 (3) % **2b** by Rietveld fitting (Figure S30), using 28 parameters (6 background, 1 zero error, 9 profile, 10 cell, 1 scale, 1 global scale factor for thermal parameters). Rietveld refinement converged to Rwp = 6.256, Rwp' = 20.292. **2a:** [*a* = 27.714 (6) Å, *b* = 9.355 (2) Å, *c* = 21.543 (6) Å, β = 117.44 (2) °, *V* = 4956 (2) Å^3]. **2b:** [*a* = 10.773 (2) Å, *b* = 10.959 (2) Å, *c* = 12.537 (1) Å, α = 71.51 (1) °, β = 76.03 (1) °, γ = 62.358 (9) °, *V* = 1235.7 (3) Å^3].

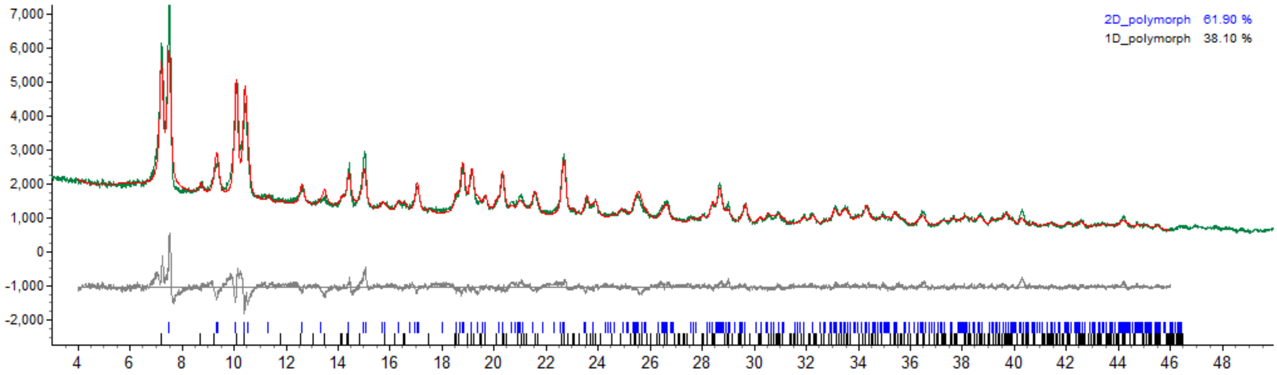

Figure S30. Observed (green) and calculated (red) profiles and difference plot [I_{obs}-I_{calc}] (grey) of the Rietveld refinement. (2θ range 4.0 – 46.0 °, d_{min} = 2.14 Å).

Phase identification: 2b exposed to 2-propanol vapour. The yellow microcrystalline product was loaded into a 0.5 mm borosilicate capillary. X-ray diffraction data were collected (λ = 1.5406 Å) at University of Sheffield, Department of Materials Science on a STOE STADI P $Cu_{K\alpha}$. Data were collected on a PSD detector with a single scan (5 < 2θ < 40 °) at a scan rate of 0.067 ° min^{-1}, using a rotating capillary. The powder pattern was indexed using the TOPAS program.[3] The pattern was compared with calculated X-ray powder patterns for polymorphs **2a** and **2b**, already established from single-crystal X-ray diffraction. The unit cell parameters for these compounds were used as a starting point for a mixed-phase Pawley refinement,[S4] employing 810 parameters (6 background, 1 zero error, 9 profile, 10 cell, 784 reflections), and resulting in final indices of fit Rwp = 3.019, Rwp' = 10.366. The starting models used for the mixed-phase Rietveld refinement,[S6] conducted using TOPAS, were the single-crystal structures of **2a** and **2b**. The model for the structure was refined using one global isotropic thermal parameter. The sample was found to contain 26.3 (3) % **2a** and 73.7 (3) % **2b** by Rietveld fitting (Figure S31), using 28 parameters (6 background, 1 zero error, 9 profile, 10 cell, 1 scale, 1 global scale factor for thermal parameters). Rietveld refinement converged to Rwp = 5.975, Rwp' = 25.096. **2a:** [*a* = 27.700 (9) Å, *b* = 9.355 (3) Å, *c* = 21.534 (7) Å, β = 117.37 (3) °, *V* = 4956 (3) Å^3]. **2b:** [*a* = 10.7645 (9) Å, *b* = 10.9798 (9) Å, *c* = 12.5310 (8) Å, α = 71.606 (6) °, β = 76.126 (7) °, γ = 62.323 (6) °, *V* = 1236.9 (2) Å^3].

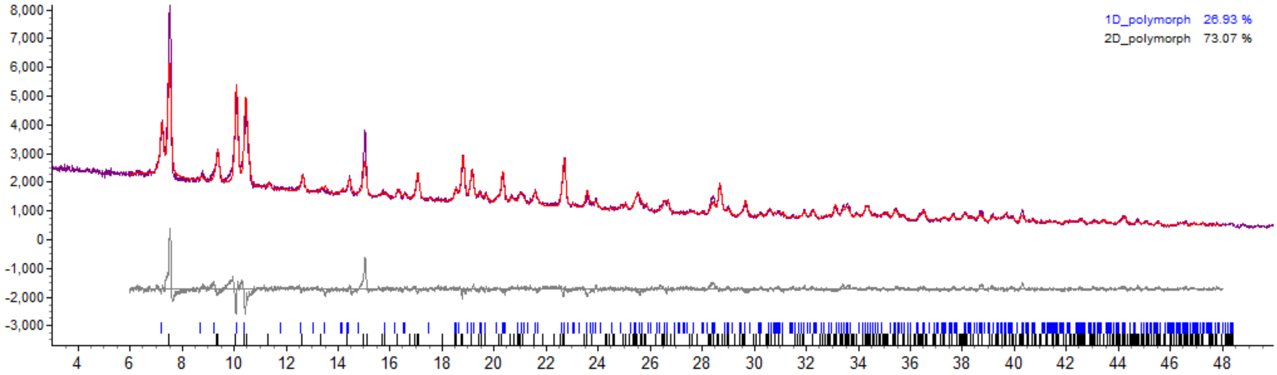

Figure S31. Observed (purple) and calculated (red) profiles and difference plot [I_{obs}-I_{calc}] (grey) of the Rietveld refinement. (2θ range 6.0 – 48.0 °, d_{min} = 2.07 Å).

Phase identification: 1-tol.tol exposed to ethanol vapour. The yellow microcrystalline product was loaded into a 0.5 mm borosilicate capillary. X-ray diffraction data were collected (λ = 0.82562(1) Å) at beamline I11 at Diamond Light Source,[S1,S2] equipped with a wide angle (90 °) PSD detector comprising 18 Mythen-2 modules. A pair of scans was conducted at room temperature, related by a 0.25 ° detector offset to account for gaps between detector modules. The resulting patterns were summed to give the final pattern for structural analysis. All data were collected at room temperature. The powder pattern was indexed using the TOPAS program.[S3] The patern was compared with calculated X-ray powder patterns for polymorphs **2a** and **2b** already established from single-crystal X-ray diffraction. The unit cell parameters of **2b**, the only phase clearly present, were used as a starting point for Pawley refinement,[S4] employing 3226 parameters (8 background, 1 zero error, 5 profile, 6 cell, 3206 reflections). Pawley refinement converged to Rwp = 5.585, Rwp' = 10.995. [a = 10.7879 (2) Å, b = 11.0064 (2) Å, c = 12.5408 (2) Å, α = 71.576 (1) °, β = 76.135 (1) °, γ = 62.282 (1) °, V = 1242.98 (4) Å^3].

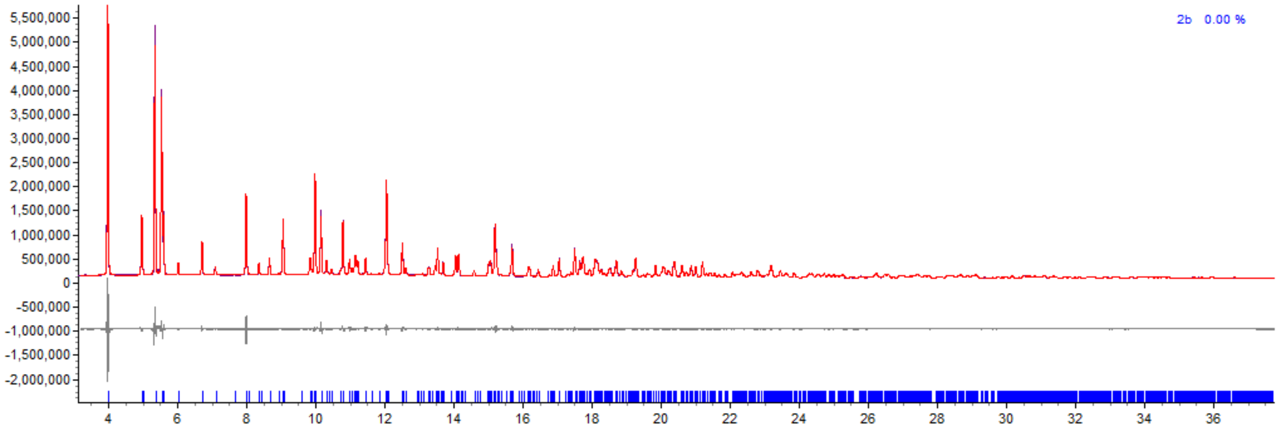

Figure S32. Observed (blue) and calculated (red) profiles and difference plot [I_{obs}-I_{calc}] (grey) of the Pawley refinement. (2θ range 3.2 – 38.0 °, d_{min} = 1.27 Å).

6. Phase identification by X-ray powder diffraction following grinding experiments

Phase identification: 2a after dry grinding. The yellow microcrystalline product was loaded into a 0.7 mm borosilicate capillary. X-ray diffraction data were collected (λ = 0.400021(9) Å) at station ID31 at the European Synchrotron Radiation Facility,[S5] using a 9-channel multi-crystal analyser crystal (MAC) detector. All data were collected at room temperature. The powder pattern was indexed using the TOPAS program,[S3] to a unit cell closely matching that of polymorph **2a**. This unit cell was used as the starting point for a Pawley refinement[S4] (Figure S33), employing 429 parameters (6 background, 1

zero error, 5 profile, 4 cell, 413 reflections). Pawley refinement converged to Rwp = 6.448, Rwp' = 14.204. [a = 27.688 (2) Å, b = 9.3675 (7) Å, c = 21.552 (1) Å, β = 117.288 (9) °, V = 4957.8 (8) Å^3]. This refinement confirmed that no conversion from **2a** had taken place.

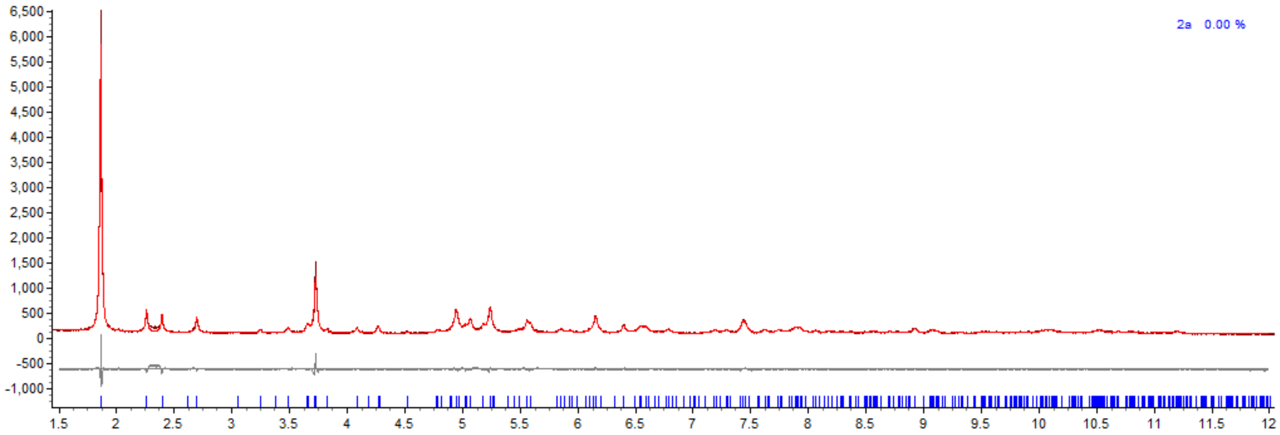

Figure S33. Observed (brown) and calculated (red) profiles and difference plot [I_{obs}-I_{calc}] (grey) of the Pawley refinement. (2θ range 1.5 – 12.0 °, d_{min} = 1.92 Å).

Phase identification, 2a after liquid-assisted grinding with acetone. The yellow microcrystalline product was loaded into a 0.7 mm borosilicate capillary. X-ray diffraction data were collected (λ = 0.400021(9) Å) at station ID31 at the European Synchrotron Radiation Facility,[S5] using a 9-channel multi-crystal analyser crystal (MAC) detector. All data were collected at room temperature. The powder pattern was indexed using the TOPAS program,[S3] to a unit cell closely matching that of polymorph **2a**. This unit cell was used as the starting point for a Pawley refinement[S4] (Figure S34), employing 345 parameters (8 background, 1 zero error, 5 profile, 4 cell, 327 reflections). Pawley refinement converged to Rwp = 8.980, Rwp' = 18.798. [a = 27.714 (1) Å, b = 9.3679 (3) Å, c = 21.5415 (8) Å, β = 117.352 (4) °, V = 4967.3 (4) Å^3]. This refinement confirmed no conversion from **2a** had taken place.

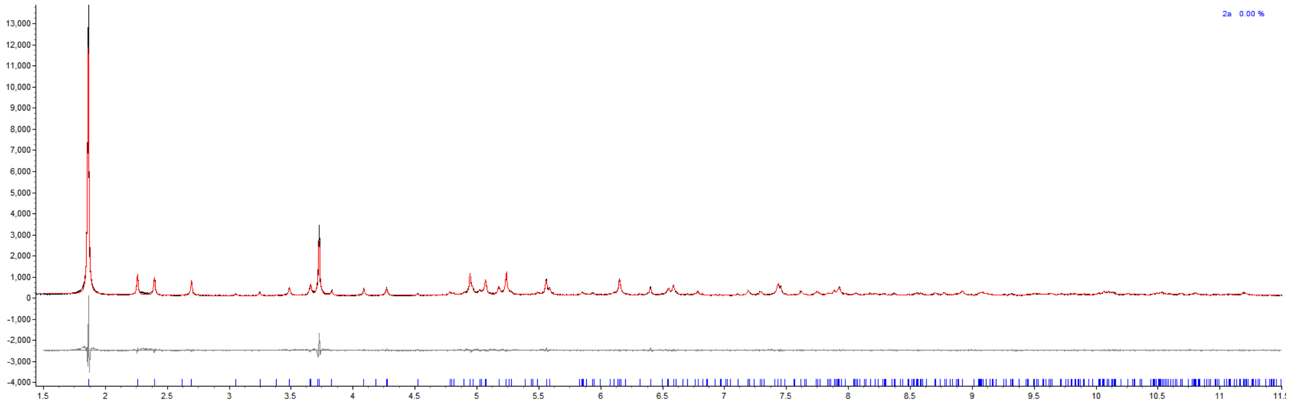

Figure S34. Observed (blue) and calculated (red) profiles and difference plot [I_{obs}-I_{calc}] (grey) of the Pawley refinement. (2θ range 1.5 – 11.5 °, d_{min} = 2.00 Å).

7. References

S1. S. P. Thompson, J. E. Parker, J. Potter, T. P. Hill, A. Birt, T. M. Cobb, F. Yuan, and C. C. Tang, *Rev. Sci. Intrum.*, 2009, **80**, 075107.

S2. S. P. Thompson, J. E. Parker, J. Marchal, J. Potter, A. Birt, F. Yuan, R. D. Fearn, A. R. Lennie, S. R. Street, and C. C. Tang, *J. Synchrotron Rad*., 2011, **18**, 637.

S3. A. A. Coelho, *TOPAS Academic, Version 4.1*, 2007; see http://www.topas-academic.net.

S4. G. S. Pawley, *J. Appl. Crystallogr*., 1981, **14**, 357.

S5. A. N. Fitch, *Res. Natl. Inst. Stand. Technol.*, 2004, **109**, 133.

S6. H. M. Rietveld, *J. Appl. Crystallogr*., 1969, **2**, 65.
